# Phylodynamic Dispersal of SARS-CoV-2 Lineages Circulating across Polish–German Border Provinces

**DOI:** 10.3390/v14050884

**Published:** 2022-04-24

**Authors:** Karol Serwin, Bogusz Aksak-Wąs, Miłosz Parczewski

**Affiliations:** Department of Infectious, Tropical Diseases and Immune Deficiency, Pomeranian Medical University in Szczecin, 71-455 Szczecin, Poland; bogusz.aw@gmail.com

**Keywords:** SARS-CoV-2, phylogeography, molecular epidemiology, transmission chains, Poland, Germany

## Abstract

Introduction: The emergence of severe acute respiratory syndrome coronavirus-2 (SARS-CoV-2) has evolved into a worldwide outbreak, with significant molecular evolution over time. Large-scale phylodynamic studies allow to map the virus spread and inform preventive strategies. Aim: This study investigates the extent of binational dispersal and dynamics of SARS-CoV-2 lineages between seven border provinces of the adjacent countries of Poland and Germany to reconstruct SARS-CoV-2 transmission networks. Methods: Following three pandemic waves from March 2020 to the end of May 2021, we analysed a dataset of 19,994 sequences divided into B.1.1.7|Alpha and non-Alpha lineage groups. We performed phylogeographic analyses using the discrete diffusion models to identify the pathways of virus spread. Results: Based on population dynamics inferences, in total, 673 lineage introductions (95% HPD interval 641–712) for non-Alpha and 618 (95% HPD interval 599–639) for B.1.1.7|Alpha were identified in the area. For non-Alpha lineages, 5.05% binational, 86.63% exclusively German, and 8.32% Polish clusters were found, with a higher frequency of international clustering observed for B.1.1.7|Alpha (13.11% for binational, 68.44% German and 18.45% Polish, *p* < 0.001). We identified key transmission hubs for the analysed lineages, namely Saxony, West Pomerania and Lower Silesia. Conclusions: Clustering patterns between Poland and Germany reflect the viral variant transmission dynamics at the international level in the borderline area. Tracing the spread of the virus between two adjacent large European countries may provide a basis for future intervention policies in cross-border cooperation efforts against the spread of the pandemics.

## 1. Introduction

The spread of the severe acute respiratory syndrome coronavirus 2 (SARS-CoV-2) has been continuously tracked since the first patient recorded in late 2019 in Wuhan, China [1,2]. Phylogenetic analyses of complete viral sequences provide near-real-time molecular surveillance of pandemic outbreaks [3] with Coronavirus disease 2019 (COVID-19) pandemics stimulating an unprecedented scale-up of sequencing efforts across Europe occurred through an expansion of the number of publicly available SARS-CoV-2 sequences [4]. Tracing of virus spread between two adjacent European Union (EU) member states can indicate trends in virus importation and domestic circulation and allow understanding of the transmission dynamics during an ongoing epidemic.

Worldwide and in Europe, the pandemic has evolved in waves (three of which were investigated in the present study: Available online: https://covid19.who.int/ accessed on 15 April 2022). The first was a mild wave observed in the spring of 2020 [5], whereas every subsequent wave has been associated with high COVID-19 rates per 100,000 individuals, significant mortality rates (Available online: https://coronavirus.jhu.edu/data/mortality (accessed on 15 April 2022)), and limitations on medical services [6,7]. More than 22,647,197 COVID-19 cases were registered in Germany as of 4 April 2022, compared to 5,978,215 incidences in Poland, meaning that these countries were within the top six most affected European Union (EU) states (Available online: https://covid19.who.int/region/euro/country/de(pl) (accessed on 15 April 2022)). During the expansion of the epidemic’s first wave, phylogenetic analyses revealed multiple independent introductions of the virus to the most populated EU member states, including variants classified as A1|19B|, A5|19 B|, B.1|20A|, B.1.1|20 B| and B.1.5|20A| [8,9,10,11]. In late March 2020, most EU countries introduced social distancing and lockdown policies [12], but this did not limit the second wave, which was largely dominated by B.1.1.177|20E.EU1| and B.1.160|20A.EU2) in Europe [13]. The expansion of newly introduced lineages followed the descending phase of COVID-19 prevalence [14]. The third wave was largely related to the outbreak of the B.1.1.7|Alpha| variant in the population [15]. Since December 2020, novel variants of concern with optimised transmissibility have emerged, some of which have evaded immune responses triggered by previous infections and vaccines [16,17,18].

To date, the number and variety of circulating transmission clusters between Poland and Germany remain unexplored. The present study reconstructs the networks of ongoing transmissions with the most common viral lineages between three West Poland regions and four East Germany states from the beginning of the pandemic to the end of May 2021. These adjacent European countries have an internal border in the Schengen area, allowing for unrestricted travel between borderline regions. Accordingly, cross-border commuting and tourism in the summer of 2020 notably might have influenced the second SARS-CoV-2 wave [14]. Precise phylogeographic investigations performed at the international level can detail the extent of transmission clusters in the inter-border areas and their influence on the introductions of novel variants and lineages into local populations related to international travel. For this purpose, to trace transmission chains of SARS-CoV-2 lineages between the border regions of the two adjacent EU countries, we time-scaled phylogenetic trees with node locations to reflect phylogeographic viral spread and illustrate the dispersal dynamics in the analysed area. Specifically, we implemented a discrete Bayesian phylogeographic method to estimate sampling heterogeneity and attempted to avoid the artefacts associated with various sampling biases. This is the first large-scale sequence-based analysis of the linkages and spread of the SARS-CoV-2 variants between Poland and Germany using molecular epidemiology methods to inform public health on the transmission dynamics related to COVID-19.

## 2. Methods

### 2.1. Sequence Data and Down-Sampling

**Figure 1:**
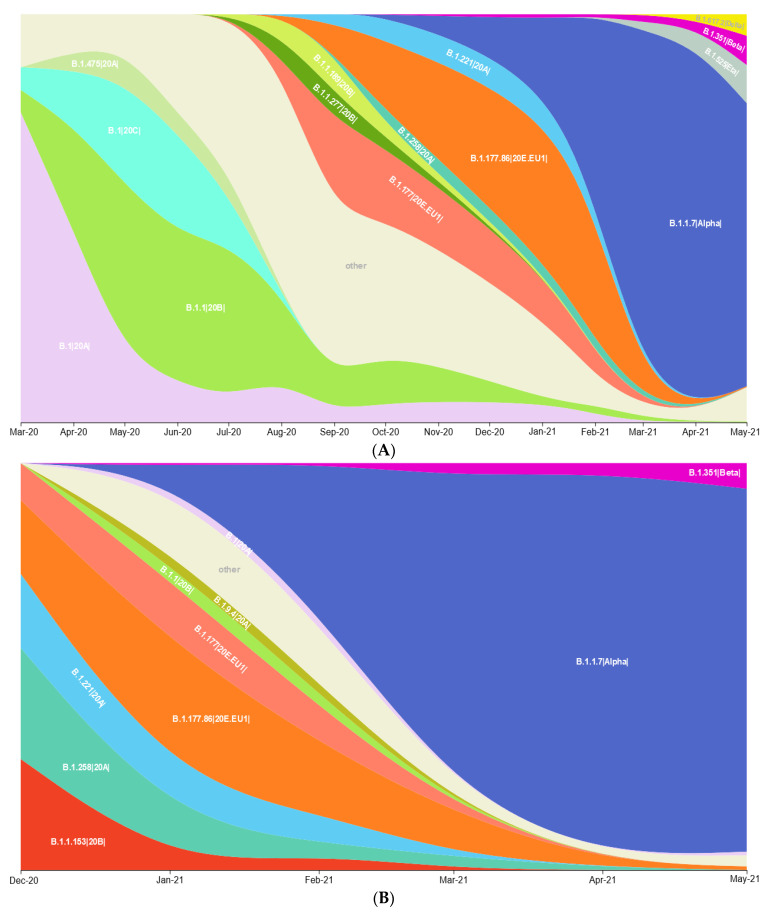

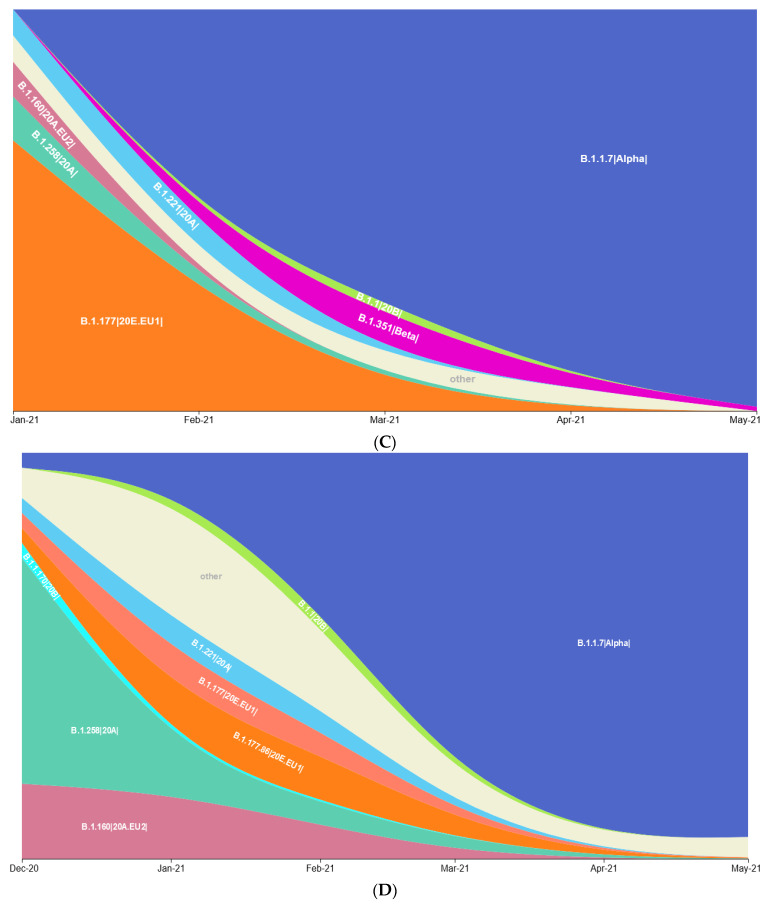

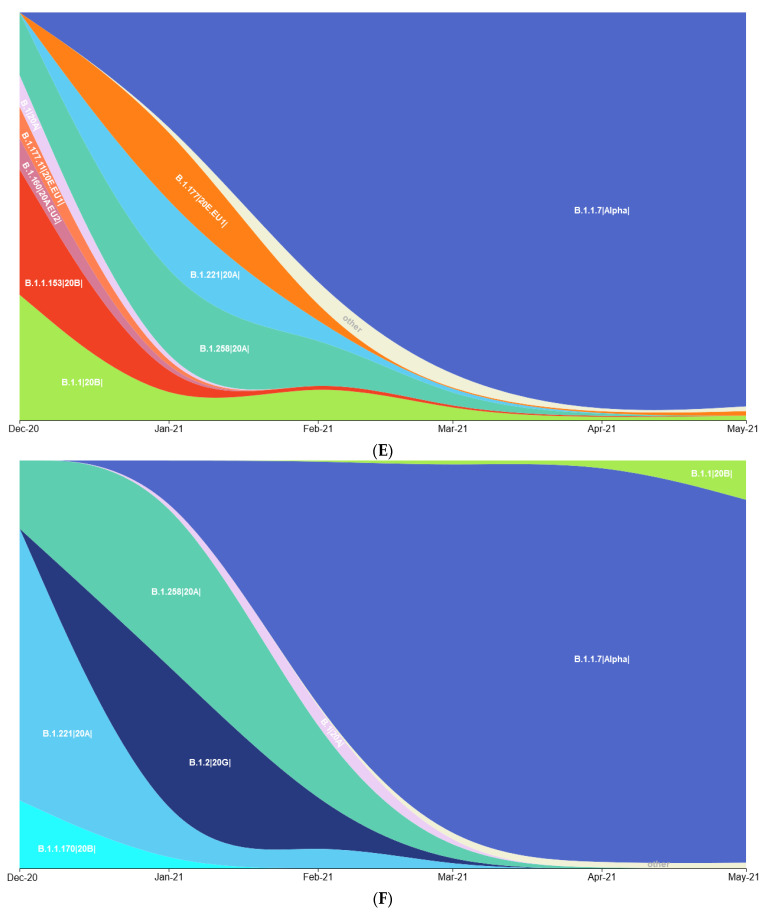

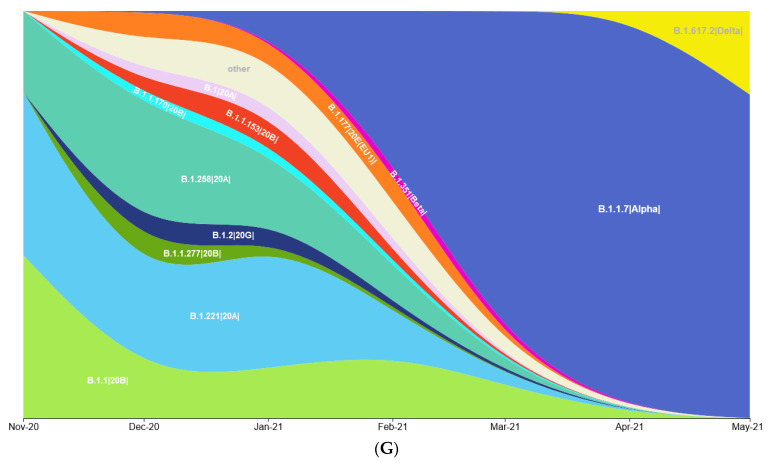
Lineage size breakdown of genomes collected monthly in each of seven investigated regions. Distribution of: (**A**) 14 major SARS-CoV-2 lineages circulating between March 2020 and May 2021 in Berlin state; (**B**) 10 major SARS-CoV-2 lineages circulating between December 2020 and May 2021 in Brandenburg; (**C**) seven major SARS-CoV-2 lineages circulating between January and May 2021 in Mecklenburg—West-Pomerania; (**D**) eight major SARS-CoV-2 lineages circulating between December 2020 and May 2021 in Saxony; (**E**) nine major SARS-CoV-2 lineages circulating between December 2020 and May 2021 in Lower-Silesia; (**F**) eight SARS-CoV-2 lineages circulating between December 2020 and May 2021 in Lubusz; (**G**) nine major SARS-CoV-2 lineages circulating between November 2020 and May 2021 in West-Pomerania.

For this study, we collected all SARS-CoV-2 regional sequences available in the Global Initiative on Sharing All Influenza Data (GISAID) database (accessed on 14 June 2021) [19], starting from 2 March 2020 and ending on 14 July 2021 (closure of data collection) for the border area between Germany and Poland. Specifically, we collected sequences from the following regions: West Pomerania (*n* = 889), Lubusz (*n* = 419), Lower Silesia (*n* = 912), Mecklenburg—West Pomerania (MWP; *n* = 1221), Brandenburg (*n* = 1365), Berlin (*n* = 4746), and Saxony (*n* = 10,442) (Germany). The Pomeranian Medical University laboratories had an essential part in sequencing efforts, delivering the 243 sequenced genomes that emerged in West Pomerania (for a total number of 889 sequences). As a result, the initial dataset included 17,774 German and 2220 Polish sequences (Figure 1A–G), and the total number of sequences from four German states was 8 times higher than in the three Polish regions. This disproportion was mostly caused by considerable sequencing efforts in Saxony.

For SARS-CoV-2 variant identification, we employed two lineage assignment tools: PANGOLIN v3.0 (Available online: https://github.com/cov-lineages/pangolin (accessed on 1 July 2021)) and NEXTCLADE v01.2.0 (Available online: https://github.com/nextstrain/nextclade (accessed on 1 July 2021)).

As the majority of sequence data from 2021 onward represent B.1.1.7|Alpha VoC, we divided the original dataset (*n* = 19,994) into two parts: the first part included all non-B.1.1.7|Alpha (*n* = 6103) sequences, with the second for B.1.1.7|Alpha (*n* = 13,891). These genome data cover the timeframes of the first, second, and third waves of the SARS-CoV-2 pandemic in selected countries [20]. We aligned the genomes from each region separately using MAFFT v.7.453 [21] and manually trimmed the alignment at the 5′ and 3′ ends, and only retained unique sequences from each region, which gives the input dataset of Alpha (*n* = 12,932) and non-Alpha (*n* = 5763) groups.

To further decrease the disparities in sampling efforts between regions, we followed a method described by P. Lemey et al. [14] and subsampled each province proportionally to the cumulative number of cases until the most recent sample date. The location with the least sampled area compared to the cumulative number of COVID-19 diagnosed infections served as a reference for down-sampling the initial number of sequences in other regions. In order to optimise the temporal and spatial coverage in each province, we marked the genomes by epi-week and sampled them as evenly as possible. Hence, the remaining number of sequences reflects an equal sampling intensity (Supplemental Figure S1A–G). We did not use the above-mentioned down-sampling procedure for Polish regional non-Alpha sequences because the beginning number of genomes was small (*n* = 425), and we wanted to retain near all available sequences. The available numbers of genomes are presented in Supplemental Data Table S3. The final analysed datasets consisted of 3359 sequences for non-Alpha lineages and 3727 isolates for B.1.1.7|Alpha VOC.

Then, we used the Nextstrain [3] focal SARS-CoV-2 sequences for Europe and the Alpha Cluster built on 14 June 2021. We sought to represent a predetermined background dataset for the B.1.1.7|Alpha and non-Alpha variants. After excluding isolates from seven investigated borderline regions from the reference dataset, we attached additional samples from other Polish and German provinces not covered in this study. To complete the background set, we employed the GISAID database and longitudinally sampled sequences from the remaining 13 Polish voivodships and 12 German states. As a result, we gathered diverse representations of 2788 genomes for non-Alpha lineages and 3169 for B.1.1.7|Alpha VoC cases originating outside the investigated borderline area. The final dataset size was 6147 sequences for non-Alpha lineages and 6896 sequences for B.1.1.7|Alpha VoC).

### 2.2. Inference of a Time-Scaled Phylogenetic Trees

We adopted the analytical workflow reported by Dellicour et al. [22] to provide an efficient phylogeographic analysis of the dispersal history of SARS-CoV-2 lineages across a specified study area. Briefly, we inferred a time-scaled phylogenetic tree of selected B.1.1.7|Alpha (*n* = 6896) and non-Alpha (*n* = 6147) sequences. To obtain a maximum likelihood phylogeny, we ran IQ-TREE v2.03 [23] under the general time-reversible (GTR) model [24] with empirical base frequencies (F) and four gamma category sites (G4) [25] that were selected as optimal for the analysed dataset using ModelFinder accuracy estimates. Inferred trees were then examined for outlier sequences using TempEst v1.5.3 [26]. After discarding 29 samples for the Alpha (*n* = 6867) variant and six for the non-Alpha (*n* = 6141) lineages, we time-calibrated each phylogeny using TreeTime v0.7.4 (TreeTime: Maximum-likelihood phylodynamic analysis; 2017–2021, Pavel Sagulenko and Richard Neher; Biozentrum, University of Basel; Evolutionary Dynamics and Biophysics, Max Planck Institute for Developmental Biology) [27]. Following the Nextstrain workflow, we specified a clock rate of 8 × 10^−4^ in TreeTime and excluded samples that differed by more than four times from a root-to-tip regression.

### 2.3. Preliminary Discrete Phylogeographic Inference

We used the discrete diffusion model [28] developed in the Bayesian Evolutionary Analysis by Sampling Trees (BEAST v.1.10.4—designed and developed by Alexei J. Drummond, Andrew Rambaut and Marc A. Suchard, Department of Computer Science, University of Auckla, 2002–2018) software package [29]. The scope of this step was to characterise the independent introduction events of the SARS-CoV-2 B.1.1.7|Alpha and non-Alpha lineages into the Polish–German inter-region. We used the time-calibrated phylogenetic trees inferred in the previous step as fixed empirical trees [30]. Only two possible ancestral locations were prespecified: the Polish–German frontier area (composed of three Polish provinces and four German states, referred to as “PL-DE-Interreg”) and “outside” the Polish–German border region [22]. Bayesian inference through Markov chain Monte Carlo (MCMC) was performed on empirical trees for one million iterations for the B.1.1.7|Alpha and non-Alpha groups separately. Runs with sampling every 1000 steps were computed, and the results were visualised and checked using Tracer (MCMC Trace Analysis Tool Version v1.7.1, 2003–2018 by Andrew Rambaut, Alexei J. Drummond, Walter Xie, Guy Baele, and Marc A. Suchard; Institute of Evolutionary Biology, University of Edinburgh; Department of Computer Science, University of Auckland; Departments of Biomathematics, Biostatistics and Human Genetics, UCL) [31]. The effective sampling size values (ESS) were 200 or more, indicating an adequate convergence. After discarding 10% of sampled trees as burn-in, the maximum clade credibility (MCC) trees were generated using TreeAnnotator v1.10.4 (part of BEAST Software Package) [31]. We applied the resulting MCC trees to describe the phylogenetic clades corresponding to independent introduction events in the studied frontier area. All phylogenetic clusters of sequences from PL-DE-Interreg were examined visually on the MCC trees to determine separate transmission lineages. According to the discrete diffusion model, we outlined the clades whose most possible location of origin was the PL-DE-Interreg region by comparing the locations assigned to each pair of nodes connected by the phylogenetic branches of MCC trees. We recognised an introduction event within the Polish–German borderline when the location indicated to a node was “PL-DE-Interreg”, and the location assigned to its parent node in the tree was an “outside” location [22,32]. Time-scaled phylogenetic trees with identified phylogenetic clades introduced in the investigated border area were delineated using functions available in the R package “seraphim”[33].

### 2.4. Discrete Phylogeographic Analyses at the Polish–German Border Regions

We performed discrete phylogeographic analysis [28] to implement the discrete diffusion model in BEAST 1.10.4 [31]. The preceding discrete phylogeographic inference identified all binational clades occurring in the seven regions of the Polish–German border area by clustering at least two sequences with known regions of origin. Every cluster fixed as a time-scaled subtree served as an empirical tree [30] for the following phylogeographic model. We incorporated the sampling region as discrete traits associated with the sampled genomes for discrete analysis according to a continuous-time Markov chain, as characterised by a matrix of asymmetrical transition rates among sampling locations [34]. The Bayesian stochastic search variable selection (BSSVS) approach was used to identify the best-supported virus transition events within the Polish–German border area [35]. Moreover, BSSVS allows for estimating the number of transitions that judiciously explain the viral diffusion process. We ran the Metropolis–Hastings algorithm for MCMC from 1 × 106 to 1.0 × 108 iterations and sampled trees at every 1000 iterations to reach sufficient ESS values as evaluated by the Tracer 1.7.1. After discarding 10% of sampled trees as burn-in, the maximum clade credibility (MCC) trees for each cluster were formed using TreeAnnotator v1.10.4. Post hoc analyses were performed using the Sprea3D tool for analysing discrete trait evolutionary histories associated with phylogenies to investigate SARS-CoV-2 spatiotemporal spread [36]. Outcomes of Markov jumps estimated by the BSSVS analyses were used to identify well-supported rates between locations in standard discrete phylogeographic reconstructions and those supported by Bayes factor (BF) values > 3 [28], which denote positive statistical support [37]. All MCC trees were visualised using FigTree v1.4.4. (Tree Figure Drawning Tool; 2006–2018, Andrew Rambaut Institute of Evolutionary Biology, University of Edinburgh). The TMRCAs distribution was calculated from the MCC trees.

## 3. Results

### 3.1. Prevalence of SARS-CoV-2 Variants between Polish–German Border Regions

**Figure 2:**
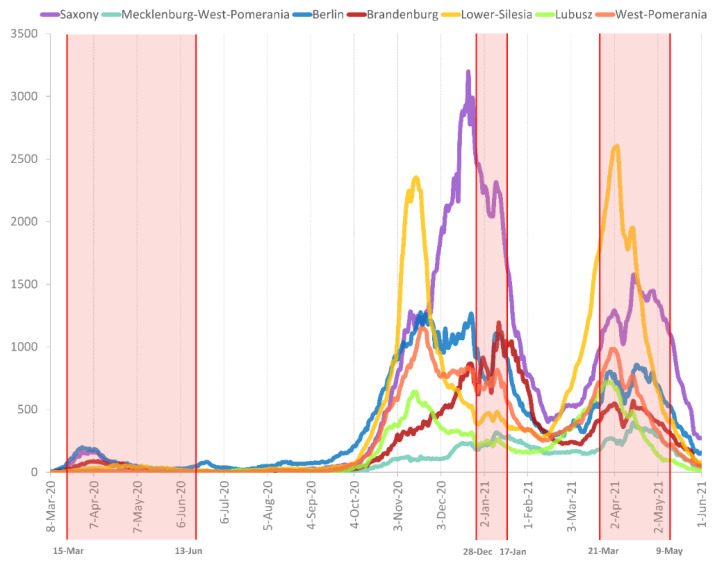
Comparison of daily confirmed COVID-19 cases in the seven studied provinces. The lines represent a 7-day rolling average. Three red rectangles range the time of most severe, restrictive measures implemented at the border—the first and the second closure introduced by Poland authorities, the third one made by the German government.

To estimate the SARS-CoV-2 genetic variant diversity and transmission dynamics between Poland and Germany, we analysed all available sequence data from the Polish and German border regions and the Berlin area through 14 July 2021. As expected, the sampling size was associated with a higher diversity of identified lineages (Supplemental Figure S2A–G), with the B.1.1.7|Alpha| lineage being the most prevalent. Interestingly, in Mecklenburg—West Pomerania (MWP) and Berlin state in Germany, as well as Lower Silesia and Lubusz in Poland, an early (>50% of sampled sequences by the beginning of February 2021) domination of the B.1.1.7|Alpha variant was observed. Moreover, in all analysed provinces, SARS-CoV-2 genetic diversity was reduced by replacing the majority of circulating lineages with B.1.1.7|Alpha VoC in the final two months of analysis (Figure 1A–G). When comparing the transmission dynamics and sequencing efforts of the three pandemic waves (Figure 2 and Supplemental Figure S1A–G) between the analysed Polish and German states, the scaling-up of the sequencing was earlier in Germany (first weeks of 2021), with an approximately 6-week delay for Poland. Interestingly, in three (Lower Silesia, Lubusz, and MWP) of the four provinces where the earliest dominance of B.1.1.7|Alpha was observed, the highest peaks in case numbers were observed in the third pandemic wave (after a replacement of circulating stains with B.1.1.7|Alpha) (Supplemental Figure S1A–G). During the second SARS-CoV-2 infection phase, B.1.177.86|20E.EU1| was ubiquitous in East Germany, and, intriguingly, we noticed only one isolate of a given strain in West Poland.

### 3.2. SARS-CoV-2 Introduction Events in the Polish–German Interregional Area

**Figure 3:**
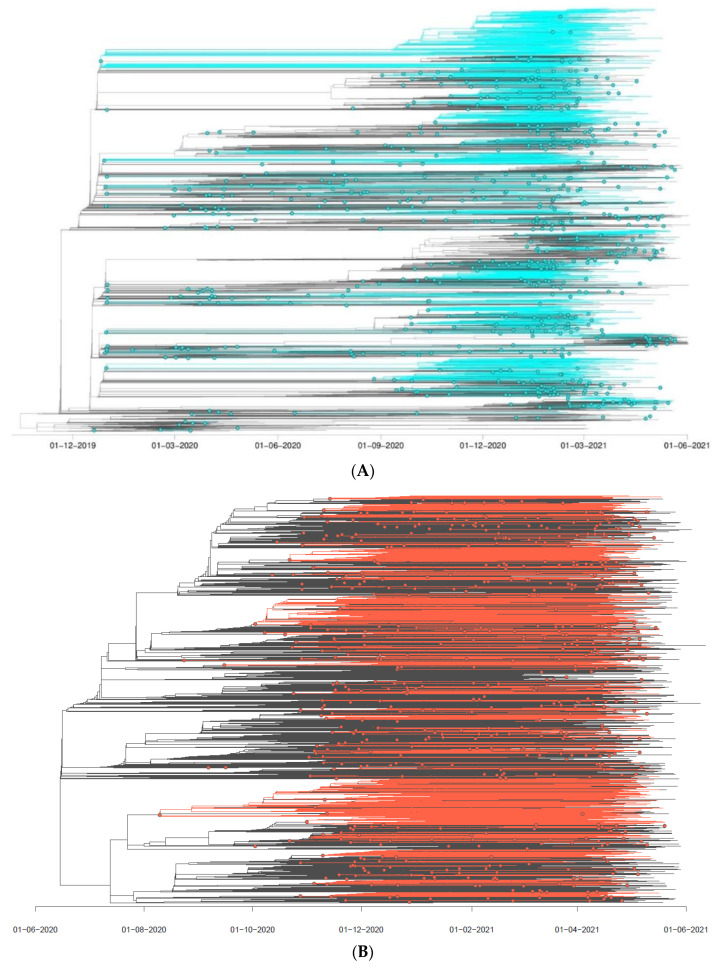
Time scaled phylogenetic tree of non-Alpha (**A**) and Alpha (**B**) sequences with identified PL-DE-Interreg clusters. A cluster is represented as a phylogenetic clade likely corresponding to a distinct introduction into the studied Polish–German frontier area. We outlined these clusters by implementing a simplistic discrete phylogeographic reconstruction along the time-scaled phylogenetic tree. Ancestral locations are identified by labelling each node in the tree as PL-DE-Interreg or outside-PL-DE-Interreg, only considering these two potential positions. On the phylogenetic tree, non-Alpha lineages circulating in the Polish–German border region are highlighted in cyan (**A**) and red (**B**). Nodes in cyan match the most ancestral node of each PL-DE-Interreg cluster.

For the analysed non-Alpha and B.1.1.7|Alpha VoC datasets, we defined the internal nodes and descending clades that corresponded to distinct SARS-CoV-2 introductions into the Polish–German border region (further referred to as PL-DE-Interreg). The discrete phylogeographic analysis allowed us to distinguish and estimate independent introduction events for non-Alpha (Figure 3A) and B.1.1.7|Alpha lineages (Figure 3B) in this region. Inference analyses indicated a minimum number of 673 lineage introductions (95% HPD interval 641–712) sampled along non-Alpha variants and a minimum number of 618 lineage introductions (95% HPD interval 599–639) for B.1.1.7|Alpha. Considering the number of sequences sampled from the Polish–German frontier area (3353) for non-Alpha lineages and (3705) for B.1.1.7|Alpha, this information reflects the relative contribution of external introductions to the local transmission chains in the investigated territory.

**Figure 4:**
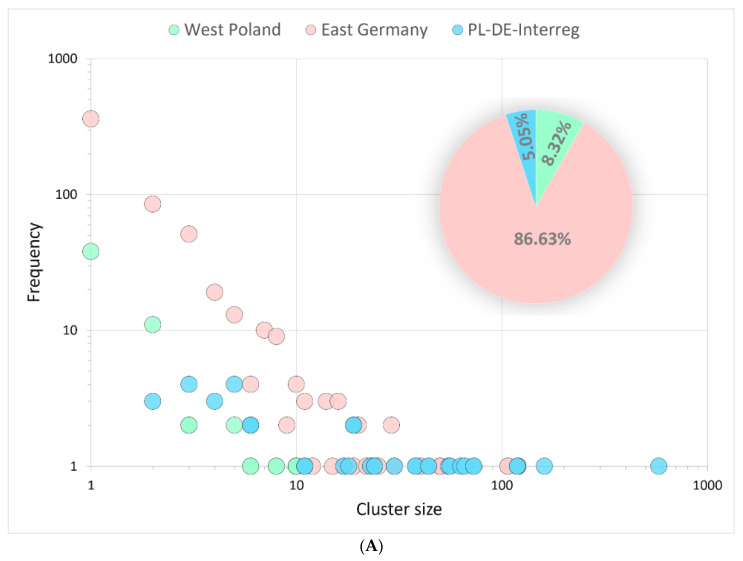

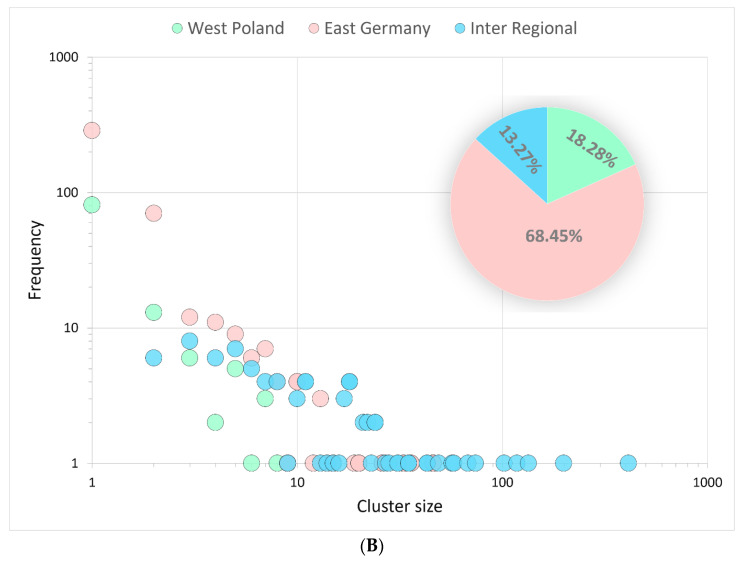
The cluster size (number of sampled sequences) distribution of the non-Alpha lineages (**A**) and Alpha VoC (**B**) circulating in the Polish–German border area. The clusters are assigned to the country of origin, containing only Polish, German, or interregional sequences (at least one from Poland and one from Germany). The pie charts show the percentage of clusters identified for a given location.

Within the estimated independent introduction events, most were singletons (59% for non-Alpha lineages and 60% for B.1.1.7|Alpha VoC) or comparatively small clades (Figure 4A,B). The major clade among non-Alpha lineages, with 581 sequences, was B.1.177.86|20E.EU1|. For B.1.1.7|Alpha VoC, the largest cluster in PL-DE-Interreg consisted of 522 sequences. If the clade contained at least two sequences sampled from a different country (Poland or Germany), it was classified as interregional. For clades with isolates sampled from only one country, the clade was defined as Polish or German.

Among the non-Alpha lineages, 34 (5.05%) were binational, 583 (86.63%) contained only German sequences, and 56 (8.32%) were exclusively Polish (Figure 4A). A notably higher frequency of international clustering was observed for binational (81, 13.11%), German (423, 68.44%), and Polish (114, 18.45%) B.1.1.7|Alpha clusters (Figure 4B), *p* < 0.001).

### 3.3. SARS-CoV-2 Circulation in the Polish–German Interregional Area

In the seven regions considered distinct and discrete locations, we identified detailed sizes and lineages for all PL-DE-Interreg clusters (Supplemental Tables S1 and S2). Furthermore, for clades containing at least 50 sequences with a minimum of 10% of the isolates originating from a single country, we employed a distinct, discrete phylogeography reconstruction fixing a time-scaled subtree as an empirical tree.

#### 3.3.1. Binational Non-Alpha Clusters

**Figure 5:**
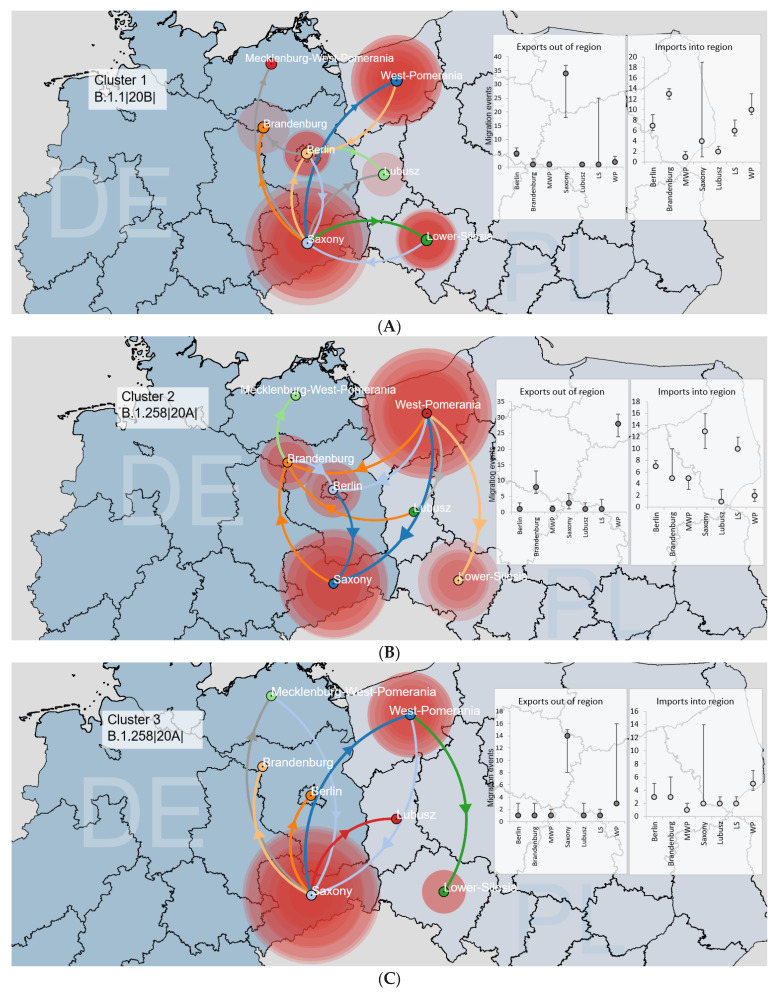

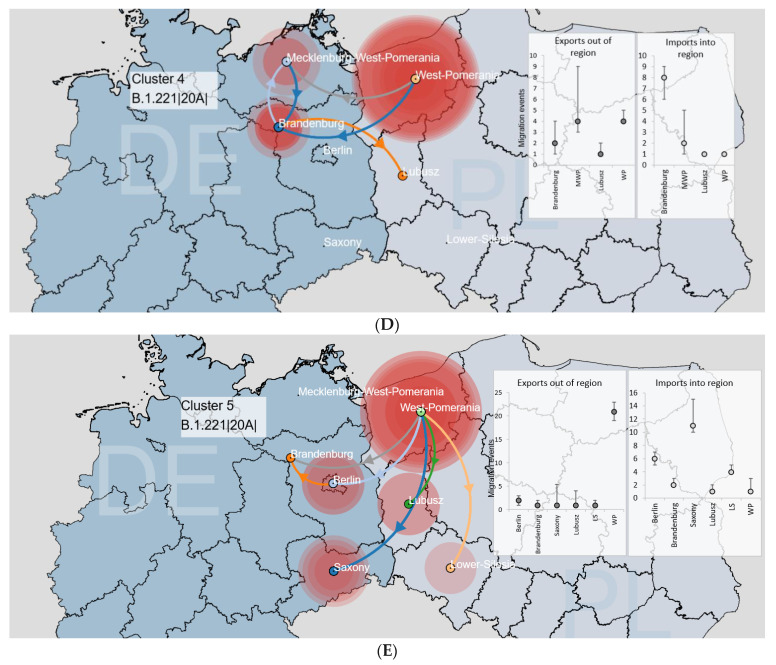
Reconstruction of the dispersal history of (**A**) Cluster 1 (B.1.1|20B|), (**B**) Cluster 2 (B.1.258|20A|), (**C**) Cluster 3 (B.1.258|20A|), (**D**) Cluster 4 (B.1.221|20A) and (**E**) Cluster 5 (B.1.221|20A) among the Polish–German border area using a discrete diffusion model. Each of the seven regions is indicated by a (centroid) circle with different colours, and inward movements to a particular location are depicted in the same colour with an arrow. The sizes of the polygons around the sampling centres are proportional to the number of viruses that maintain a specific area. The charts show a median number of transitions out of and into each region, as inferred using a Markov jumps estimation with the 95 % HPD intervals shown as lines. All migration events between locations are supported by Bayes Factor values ≥ 3, except for lines indicated in grey.

We thoroughly analysed the circulation dynamics of the five major circulating non-Alpha clusters in the area to reflect the transmission dynamics between the locations. Cluster 1, identified as B.1.1|20B|, contained 161 sequences (57.76% were of German origin) (Figure 5A and Supplemental Figure S3A). The central transfer hub for the virus was Saxony, with lineage dispersal events in four regions (Brandenburg, West Pomerania, Berlin and Lower Silesia). The time to the most recent common ancestor (TMRCA) was estimated on 20 October 2020 (Supplemental Figure S4A). Two further clusters (Clusters 2 and 3) were of the B.1.258|20A|lineage, containing 119 and 73 sequences, respectively (Figure 5B,C and Supplementary Figure S3B,C). The primary transfer cores were West Pomerania and Saxony, respectively, with the variant dispersed into Saxony and Lower-Silesia for Cluster2 and West Pomerania for Cluster3. The TMRCAs for both clades were estimated on 4 and 21 September 2020 (Supplemental Figure S4B,C). The last two large Polish–German clades were established via lineage B.1.221|20A|, largely of Polish origin (65.15% for Cluster 4 and 56.36% for Cluster 5; Figure 5D,E and Supplementary Figure S3D,E), with West Pomerania as the central source point and TMRCA occurring between September and October 2020 (Supplemental Figure S4D,E). Most isolates of the identified non-Alpha clusters were collected between January and March 2021. Additionally, we calculated the detection lag (described as the number of days between the inferred transmission lineage time to TMRCA and its first sampled sequence [38]) with a median of 45 days (IQR: 56–67 days).

Interestingly, we identified a large (B.1.1.170|20E.EU1| variant) interregional cluster of 581 sequences in the Berlin, Brandenburg, and Saxony regions, with only one transmission link to Poland (in West Pomerania) (Supplemental Figure S5).

#### 3.3.2. Binational B.1.1.7|Alpha Clusters

**Figure 6:**
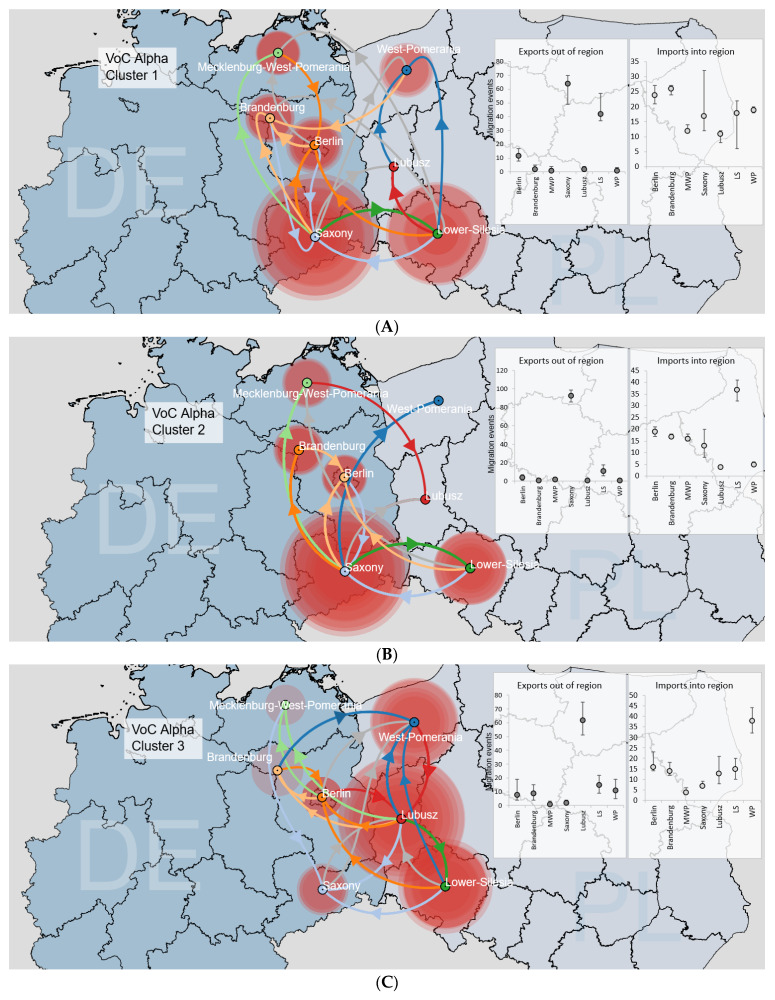

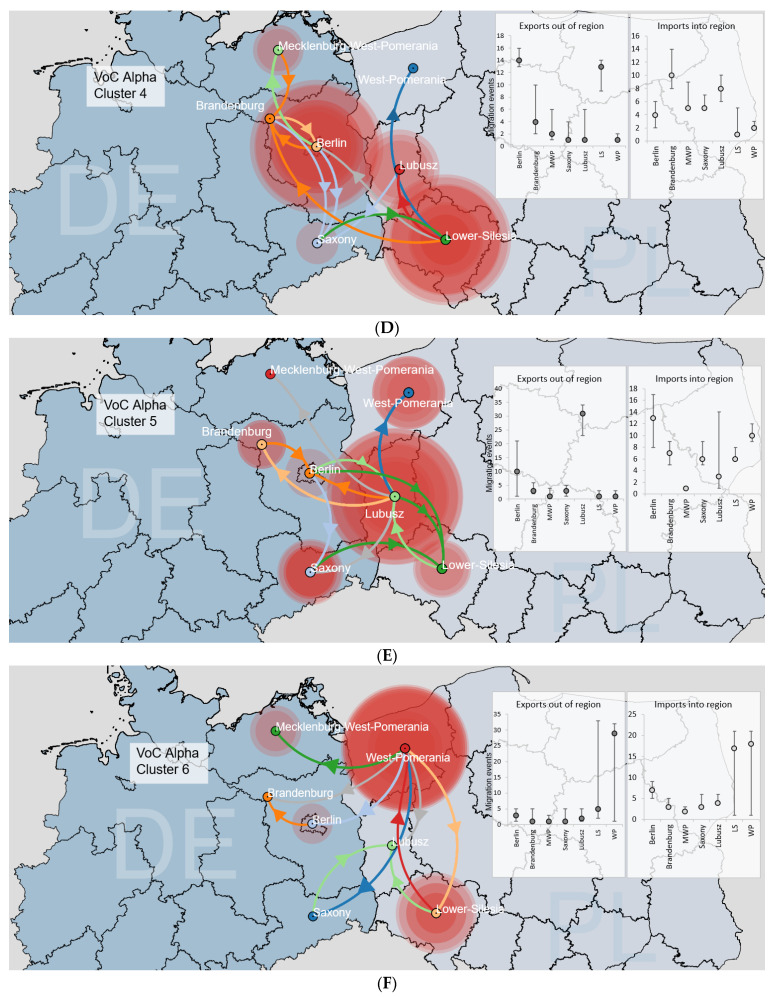

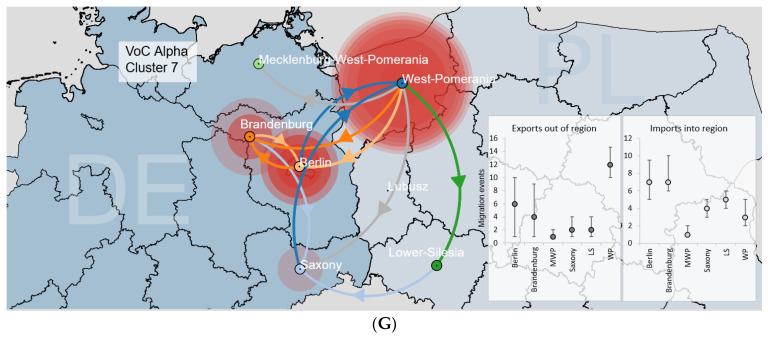
Reconstruction of the dispersal history of the nine largest VOC Alpha Clusters (**A**–**G**) among the Polish–German border area using the discrete diffusion model. Each of the seven regions is indicated by a (centroid) circle with different colours, and inward movements to a particular location are depicted in the same colour with an arrow. The sizes of the polygons around the sampling centres are proportional to the number of viruses that maintain a specific area. The charts show a median number of transitions out of and into each region, as inferred using a Markov jumps estimation with the 95% HPD intervals shown as lines. All migration events between locations are supported by Bayes Factor values ≥ 3, except for lines indicated in grey.

Similarly, we conducted discrete phylogeographic analyses of B.1.1.7|Alpha VoC. Seven large B.1.1.7|Alpha clusters were identified in the area (Figure 6A–G). Four clusters expanded from West Poland (Clusters 3, 5, 6 and 7), with the remaining clades being primarily of German origin (Clusters 1, 2 and 4; Supplemental Figure S6A–G). Central transmission hubs included Lower Silesia (Clusters 1 and 4), Saxony (Clusters 1 and 2), Berlin (Cluster 4), and West Pomerania (Cluster 6). Interestingly, B.1.1.7|Alpha Clusters had multi-center locations for the virus sink destinations, located mainly in Silesia (Clusters 2 and 6), West Pomerania (Clusters 3 and 6), Brandenburg (Clusters 1, 4 and 7), Berlin (Clusters 1, 5 and 7). The TMRCA for each clade indicated a range of introduction events between 5 November 2020 and 19 January 2021, with the majority of samples assembled from February to May 2021 (Supplemental Figure S7A–G). The calculated detection lag of B.1.1.7|Alpha transmission clusters was 42 days (IQR: 38–46 days). The largest clade was comprised of 484 sequences (for details, see Supplemental Table S2).

## 4. Discussion

In this study, we used a large dataset of binational sequences and robust phylodynamic and phylogeographic analyses to detail the importance of transmission networks between West Poland and East Germany from March 2020 to June 2021. Selected regions are historically and economically linked by common international commuting for work and local bilateral trade [39]. No similar international analysis for the transmission links between Western and Central European countries was performed before. We observed a decrease in SARS-CoV-2 genetic diversity related to the domination and replacement of most lineages by the B.1.1.7|Alpha variant of concern in early 2021, with the increase in the number of binational transmissions following the domination of this VoC. Moreover, we reconstructed detailed infection dynamics for the major clusters identifying regional transmission hubs.

The emergence of B.1.1 in Europe around February 2020 implicates the epidemic’s first wave and locations where B.1.1 was prevalent, presumably acting as importation spots [40]. Observed B.1.1 Cluster 1 (the largest non-Alpha group) with TMRCA calculated in mid-October 2020 suggests import of the virus from the established European lineage or a section of a much bigger clade restricted by the number of available sequences at the beginning of the first wave.

During the second pandemic wave, viral diversity was notably more extensive, with multiple variants being observed, including the most common German B.1.177.86|20E.EU1| lineage (descended from B.1.177|20E.EU1), which emerged in the Summer of 2020 and became the most prevalent in the Autumn of 2020 in numerous West European countries [13]. Increased frequency of infections with this variant was first identified in Spain with subsequent pan-European spread contributed by onward transmission events largely fuelled by the United Kingdom following holiday travel of 2020, but this lineage was not associated with increased transmissibility [14]. Interestingly, only one B.1.177.86|20E.EU1| sequence was identified in Poland (region of West Pomerania), so there were no substantial binational transmission clusters for this lineage, but the timeline of this variant European dominance coincided with limited mobility between border provinces [41]. Of note, in PL-De-Interreg, the two subsequent prevalent lineages, B.1.258|20A| and B.1.221|20A|, from the second wave had been circulating extensively, and it appears to be congruent with patterns of incidence observed in West Poland [11] and East Germany [42].

In the subsequent months of the pandemic, domination of B.1.1.7|Alpha VoC was associated with the observed reduction in the number of circulating lineages and, therefore, viral genetic diversity was most likely associated with its increased transmissibility [43]. Early domination of B.1.1.7|Alpha was observed in the three analysed borderline regions (Lower Silesia, Lubusz and MWP), resulting in the highest incidence of infections in these regions following the introduction of VoC. These peaks in incidence may also be associated with higher population susceptibility due to a lower previous number of infections during the first and second COVID-19 waves in identified regions.

Importantly, following the observed replacement of most circulating lineages with B.1.1.7|Alpha VoC, the number of binational German–Polish clusters notably propagated (from 5.05% to 13.27%), thereby increasing the significance of international transmission events for virus spread. Notably, the lower number of binational transmissions coincides with the period of limitation in citizen mobility, including German–Poland border closure in the early COVID-19 pandemics, which likely resulted in more contained, national pandemics for the non-Alpha variants. From 15 March 2020 until 13 June 2020, Polish authorities had closed country borders to international travel (Available online: https://www.gov.pl/web/coronavirus/travel (accessed on 15 April 2022)). Later, the German government introduced restrictions for Poland citizens as inhabitants of a “high-risk region” from 24 October 2020 and “high-incidence area” on 21 March 2021. On 9 May 2021, the country’s status was reverted to a “normal” risk area (Available online: https://www.rki.de (accessed on 15 April 2022)). Lockdown measures were abolished or loose prior to the domination of the B.1.1.7|Alpha variant, reflected by an increased number of international transmission and import/export events in the current phylogeographic analysis.

Phylogeographic analyses indicated complex transmission dynamics for the identified clades, with central hubs and bidirectional transmission events occurring even in distant regions, thus reflecting high human mobility [28,44,45]. A high proportion of observed singletons suggests that onward transmission of the introduced virus was uncommon despite multiple independent introduction events. The estimates of the relative contribution of external introductions in establishing regional transmission chains in PL-DE Interreg gave similar observations to the one noted in New York State (116 introductions in 828 sampled genomes) [46] and Finland (42 introductions of 333 genomes) [47]. Insights from regions sampled at very high densities, such as the United Kingdom, show that the majority of introductions lead to small, transient, dead-end transmission lineages, whereas a small number of introductions lead to larger and longer-lasting transmission lineages [38]. The larger clades identified in our study suggest that virus transmission events, especially for the B.1.1.7|Alpha were frequent between neighbouring and well-connected provinces of Germany and Poland, especially Saxony and Lower Silesia.

Study limitations: Our analysis may be limited by various factors. The sample size of polish origin restricts our ability to infer further details about local virus transmission dynamics from sequence data alone. Moreover, the broad range of lag times between the inferred TMRCAs and the earliest sampled sequence per transmission lineage suggests that a considerable number of transmission lineages are not detected immediately after being introduced to the country, reducing our capacity to identify a potential port of entry and delaying the possibility of responding rapidly to new seeding events. Additionally, the size of the clusters reflects a minimal number of sequences in each group because it was restricted by the number of inferred genomes and the down-sampling procedure.

## 5. Conclusions

Identifying circulating binational clusters may assist in tracking pandemic networks and inform decisions related to distance measures and vaccination strategies [48]. In regions with early emergence and replacement of the circuiting clades by the highly transmissible variant of concern, the pandemic wave was extensive and associated with a singular dominant VOC [38]. Phylogeographic and phylodynamic reconstructions allow for tracing the evolution of the virus and reflect lineage-specific differences in transmissibility and infections. Interrupting and ultimately stopping transmission chains is not only important to minimise virus spread within populations, but also to reduce the accumulation of mutations and evolution of the new variants, with the studies on viral transmission dynamics being key to reflecting real-life transmission events and mobility. Our findings highlight the importance of molecular surveillance of frontier areas as regions where large clusters are circulating.

## Data Availability

The sequences used in this work have been deposited in the GISAID. Supplemental Table S3 lists all the appropriate identification numbers.

## Supplementary Materials

The following supporting information can be downloaded at: https://www.mdpi.com/article/10.3390/v14050884/s1, **Figure S1A–G**. Histograms of weekly COVID-19 cases in each analyzed region (mapped to left *y*-axis); together with fluctuations of weekly sequencing efforts (shown as a green line mapped to the right *y*-axis), and the number of sequences used as a dataset in this study (presented as the purple line mapped to the right *y*-axis). EpiWeek marks the following weeks of the epidemic in 2020 and 2021. **Figure S2A–G**. Partition of genomes in each of seven regions into specific lineages. **Figure S3A–E**. Frequency and distribution of main non-Alpha clusters circulating in Polish-German borderland area over time. **Figure S4A–E**. Time-resolved maximum clade credibility phylogeny of the main non-Alpha clusters among Polish-German borderland area, with province location indicated. **Figure S5**. Reconstruction of the dispersal history of the largest identified non-Alpha Cluster among Polish-German borderline area under discrete diffusion model. **Figure S6A–G**. Frequency and distribution of the primary Alpha clusters circulating in Polish-German borderland area over time. **Figure S7A–G**. Time-resolved maximum clade credibility phylogeny of the Alpha clusters among Polish-German borderland area, with province location indicated. **Table S1**. List of non-Alpha Clades identified in the Polish-German frontier area. **Table S2**. List of VOC Alpha Clades identified in the Polish-German frontier area. **Table S3** lists all the appropriate identification numbers.
