# Supplementary material for: Phylodynamic Dispersal of SARS-CoV-2 Lineages Circulating across Polish–German Border Provinces
Source: Viruses. 2022 Apr 24;14(5):884. doi: 10.3390/v14050884 (PMC9143188; doi:10.3390/v14050884)
Supplement: Supplementary file 1 [file viruses-14-00884-s001.zip › viruses-1655018-supplementary.pdf]

Supplemental data

Supplemental Figure S1A–G

For all analysed provinces three epidemic waves may be observed. First began in March-2020 and lasted until mid-April-2020. The epidemiological profile of this phase in the seven provinces that were the scope of this analysis was very similar. The second wave started in October-2020 and terminated in mid-February. For the three Polish regions, the peak incidence was in mid-November, with the highest number of cases, 2,335 (17 November 2020) - for Lower Silesia. In German states (except Berlin), the frequency profile was shifted by more than a month with an extended peak of 3,198 (22 December 2021) for Saxony. The rate recorded in Berlin remained at a high (> 1000 per day) level until January 2021, subsequent a decline was recorded for two weeks, next to a secondary peak emerged. The last noted phase of the epidemic began in February-2021 and has dropped in May 2021. The development of the third period of the epidemic was broadly equivalent in eastern Germany and western Poland, with the highest infection rate of 2,693 on 4 April 2021 for Lower Silesia. Except for Berlin state and individual cases in the other studied areas, molecular surveillance was scaled at the end of the second pandemic wave, i.e., December 2020. Sequencing efforts in Germany were substantially more significant than in Poland.

Supplemental Figure S1 A–G. Histograms of weekly COVID-19 cases in each analyzed region (mapped to left y-axis); together with fluctuations of weekly sequencing efforts (shown as a green line mapped to the right y-axis), and the number of sequences used as a dataset in this study (presented as the purple line mapped to the right y-axis). EpiWeek marks the following weeks of the epidemic in 2020 and 2021.

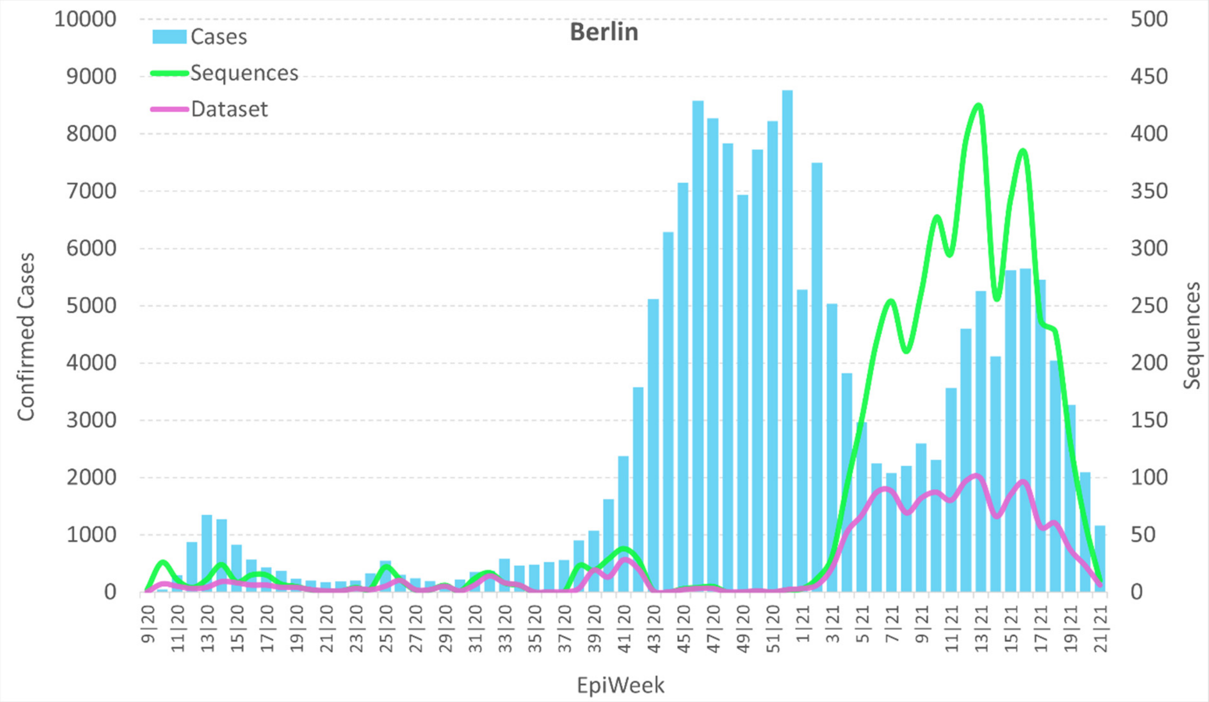

Supplemental Figure S1A

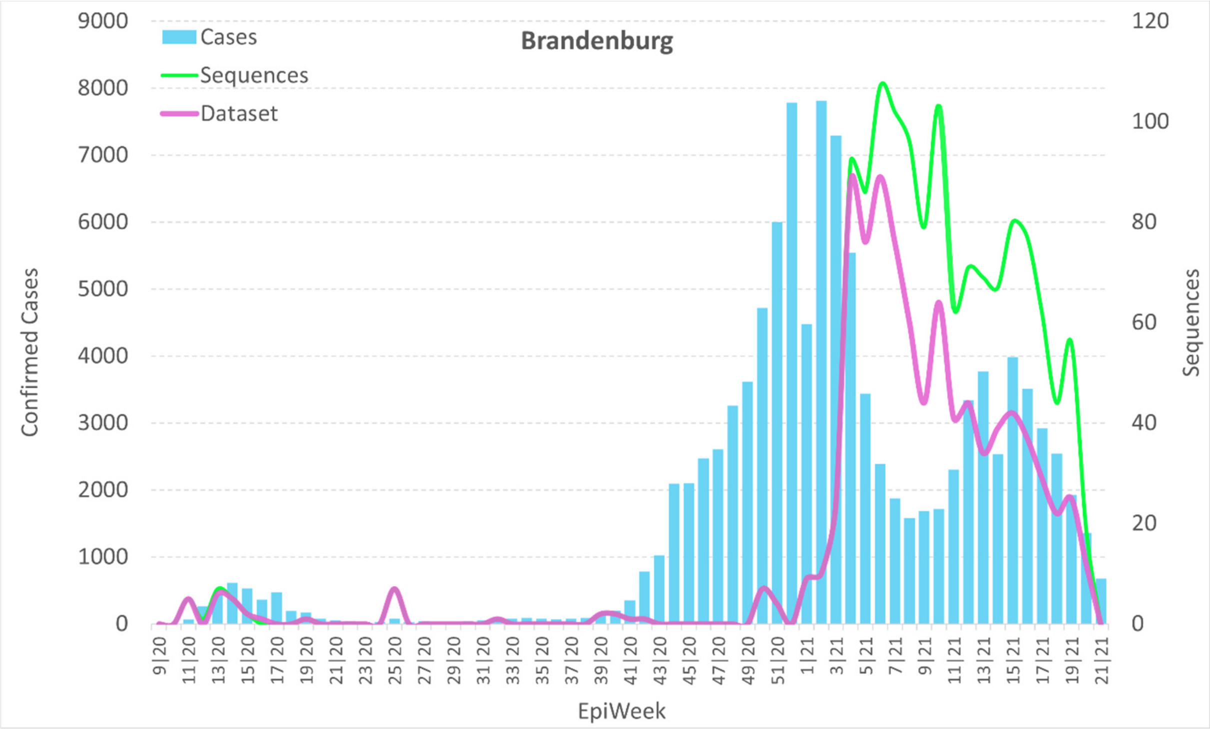

Supplemental Figure S1B

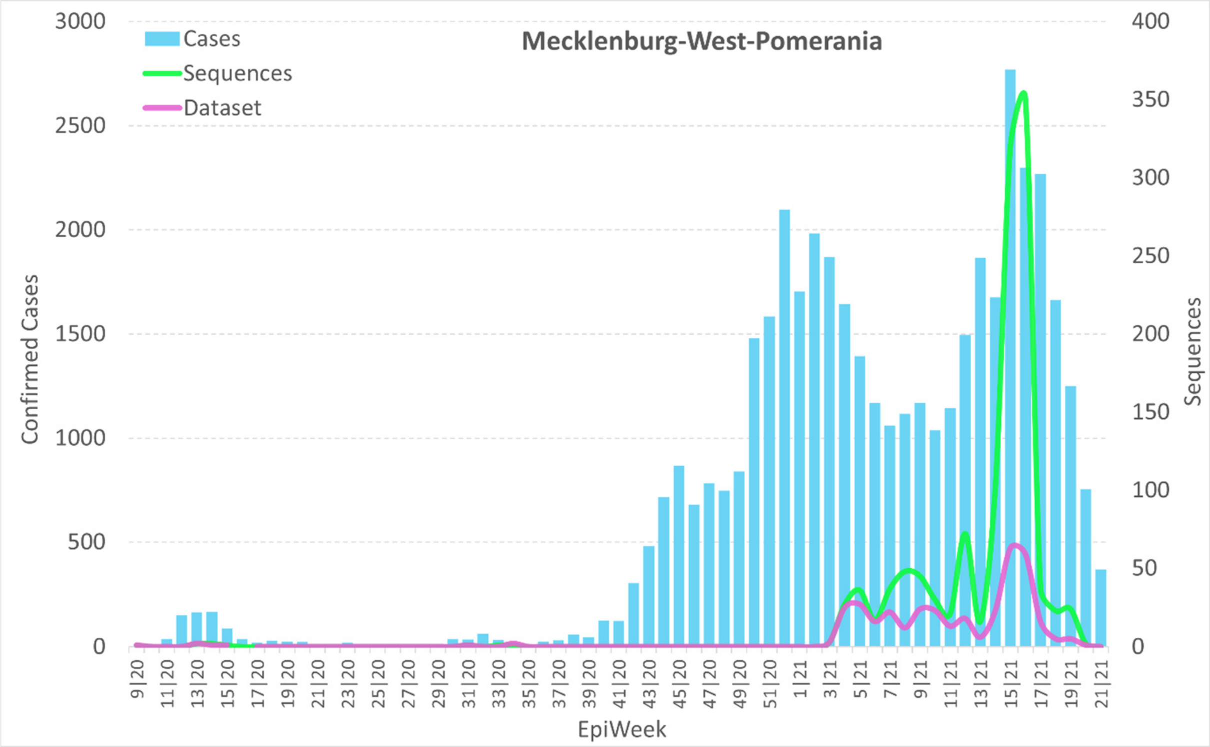

Supplemental Figure S1C

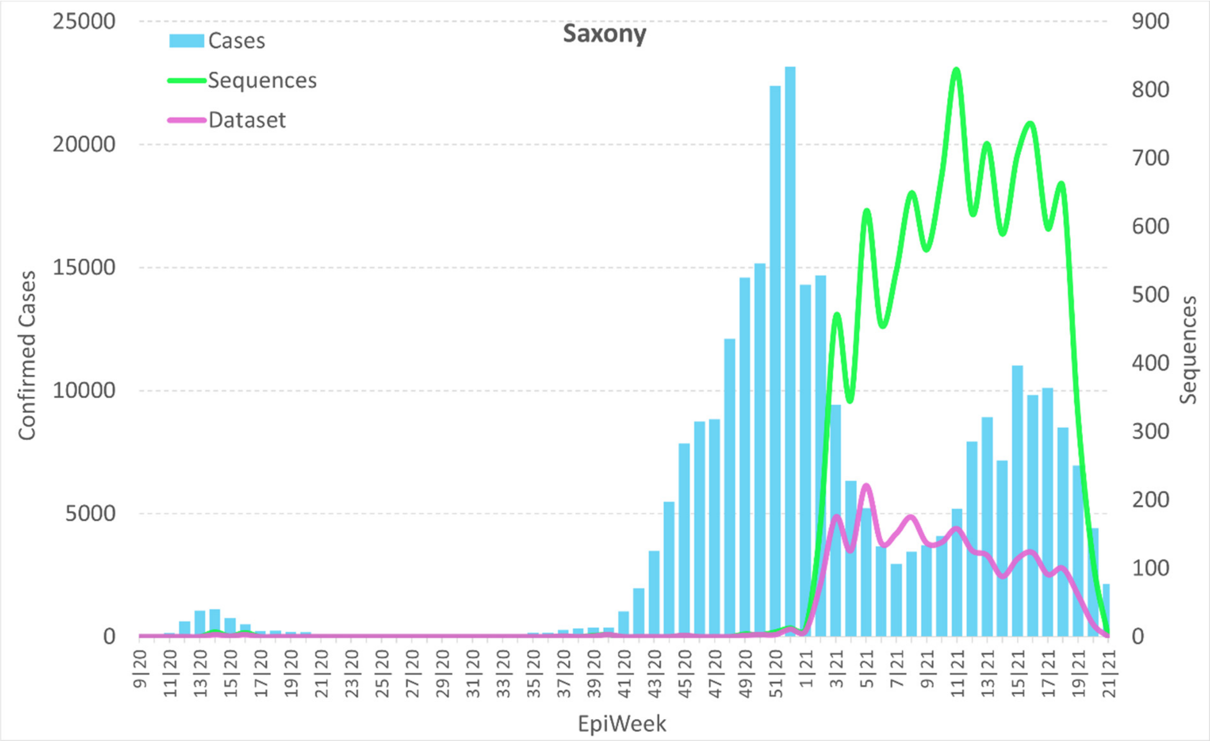

Supplemental Figure S1D

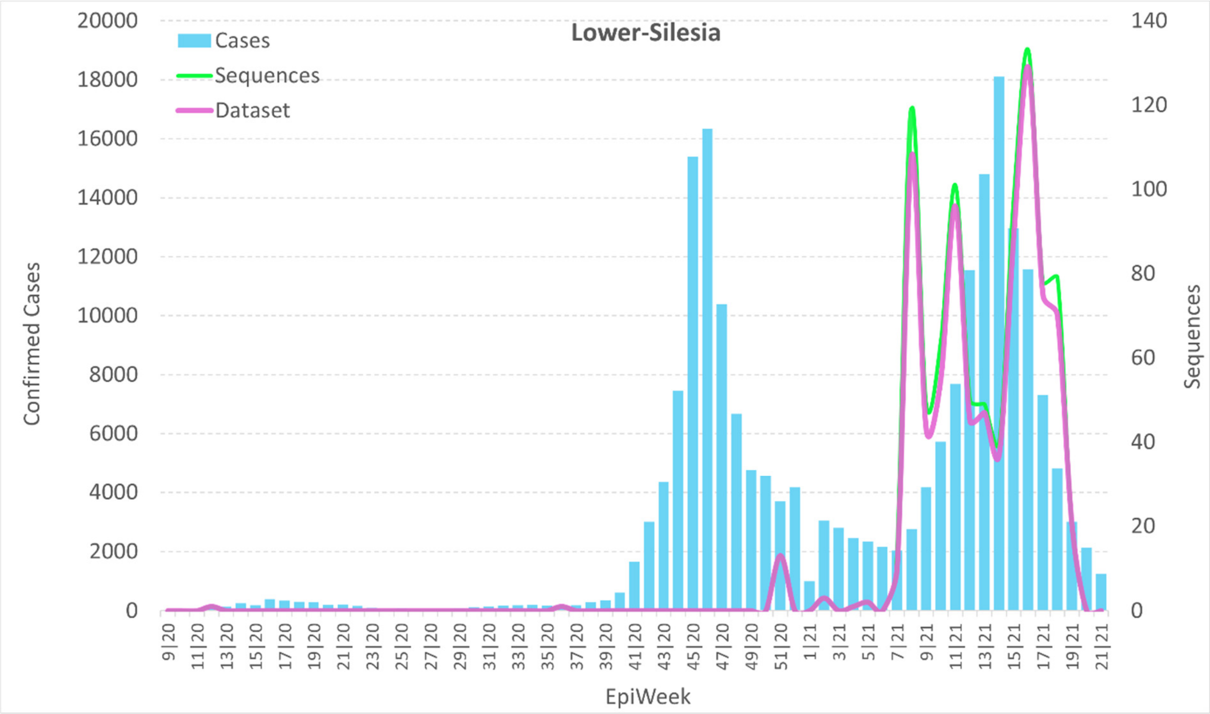

Supplemental Figure S1E

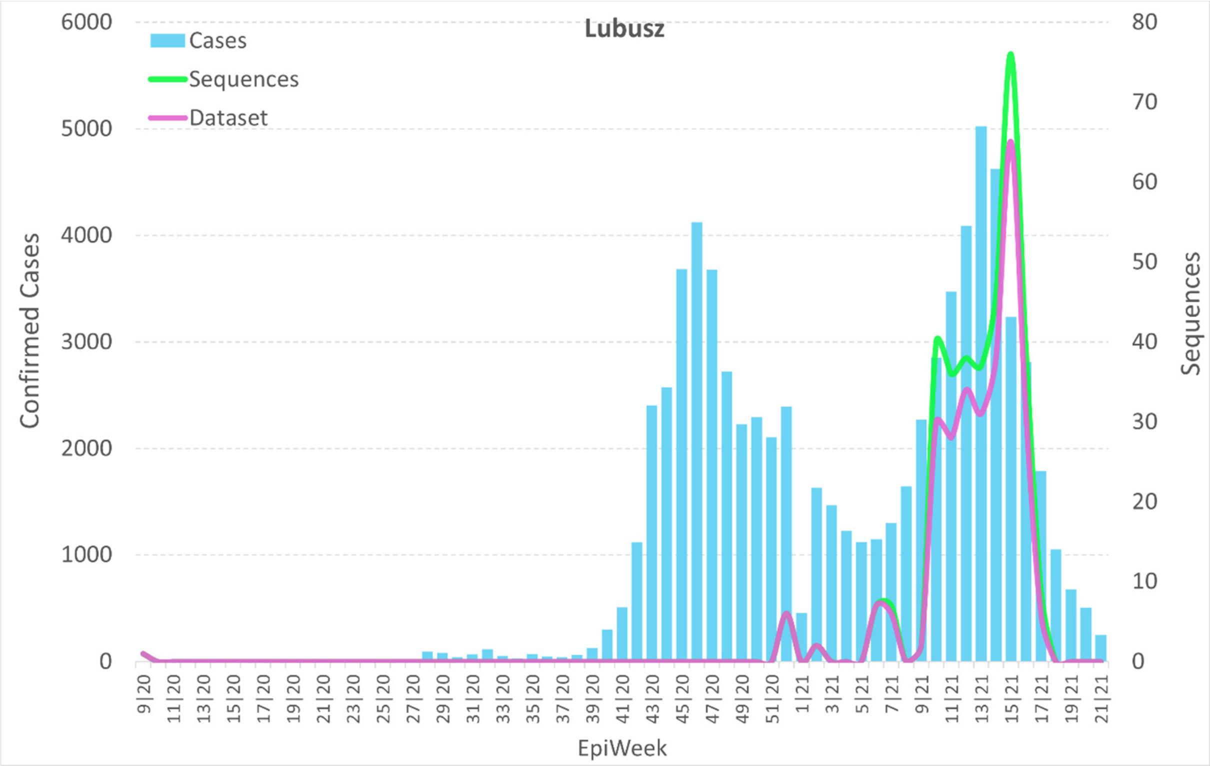

Supplemental Figure S1F

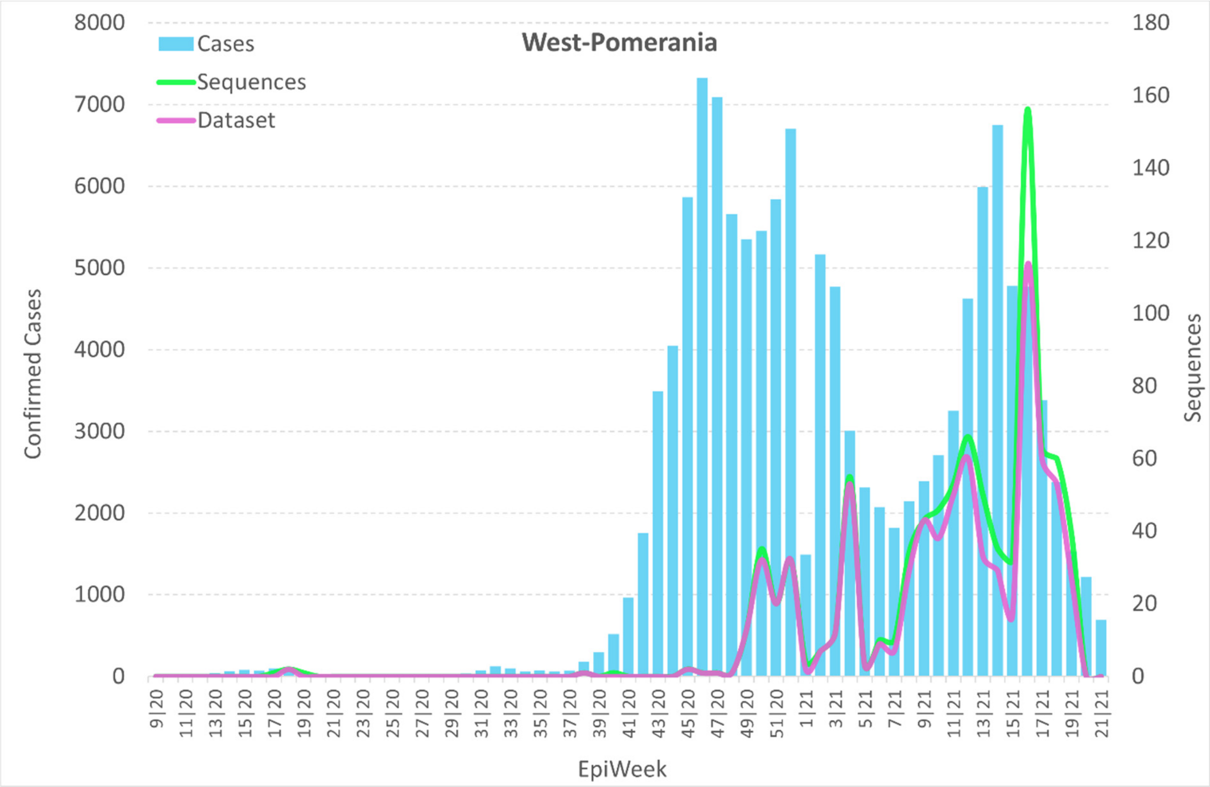

Supplemental Figure S1G

Supplemental Figure S2A–G

Regionally, in the analysed German provinces, except for the most common B.1.1.7|Alpha| variant, the second-most common lineage was B.1.177.86 |20E.EU1|e. In contrast, in the Western Poland the abundant non-B.1.1.7 variants were B.1.1|20B| in Lower-Silesia and Lubusz and B.1.221|20A| in West-Pomerania. Together with variant B.1.258|20A| these three lineages constitute the most numerous non-Alpha variants in Western Polish states.

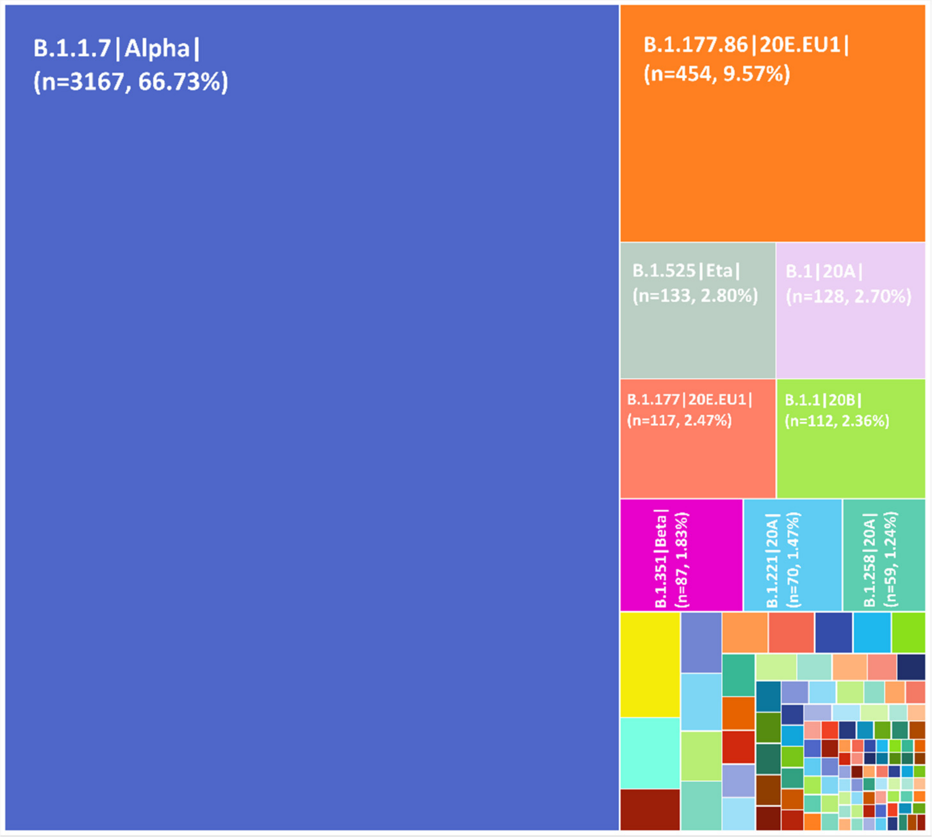

Supplemental Figure S2A. Partition of 4746 Berlin state genomes into 120 identified lineages. The size and color of each rectangle reflect the share of a given variant in the studied region. For the nine largest (>1%) lineages, the name, number of isolates, and percentage share of total sequences were shown.

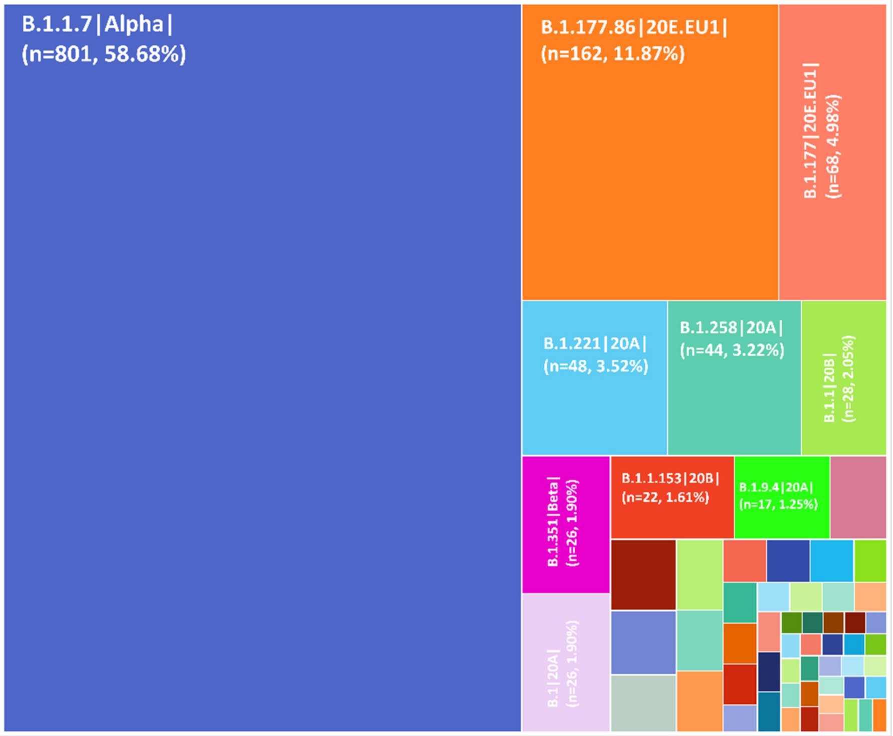

Supplemental Figure S2B. Partition of 1365 Brandenburg genomes into 59 identified lineages. The size and color of each rectangle reflect the share of a given variant in the studied region. For the ten largest (>1%) lineages, the name, number of isolates, and percentage share of total sequences were shown.

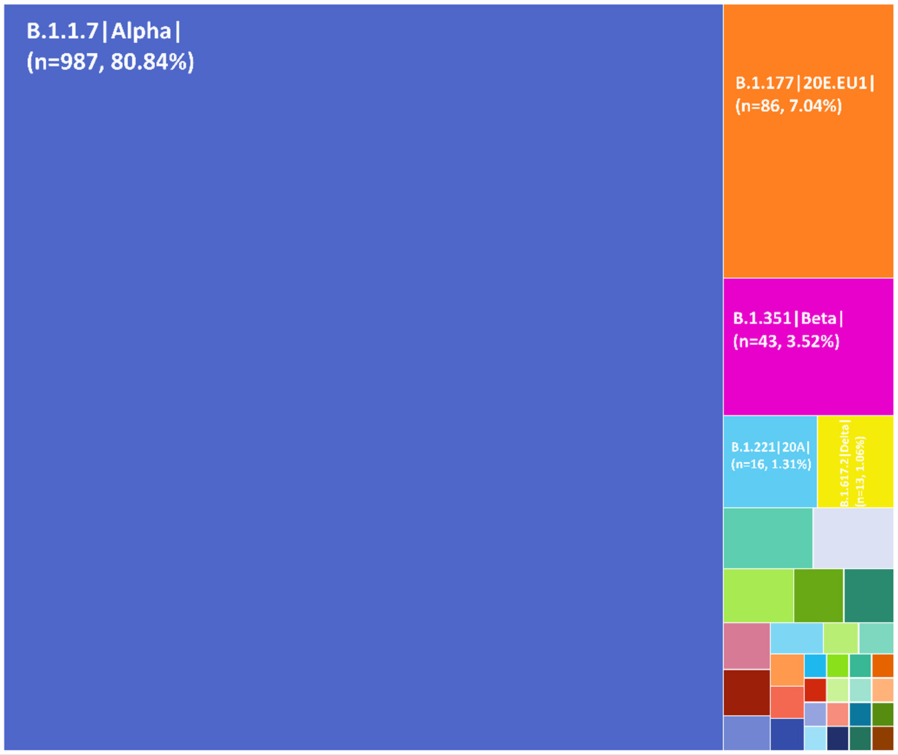

Supplemental Figure S2C. Partition of 1221 Mecklenburg-West-Pomerania genomes into 35 identified lineages. The size and color of each rectangle reflect the share of a given variant in the studied region. For the five largest (>1%) lineages, the name, number of isolates, and percentage share of total sequences were shown.

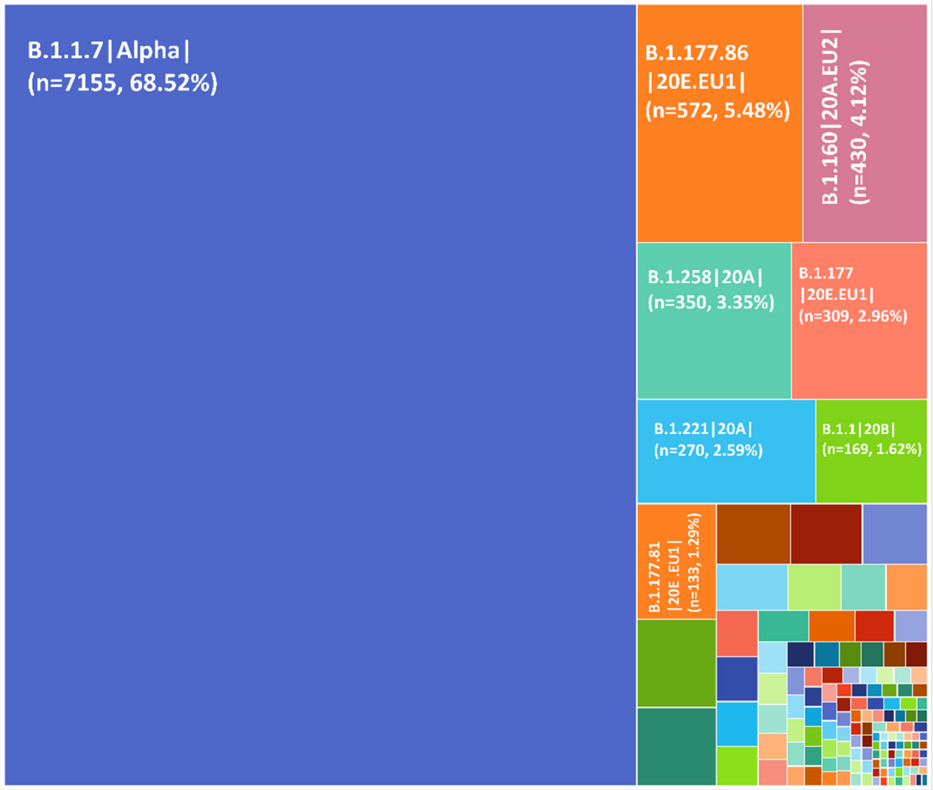

Supplemental Figure S2D. Partition of 10422 Saxony genomes into 140 identified lineages. The size and color of each rectangle reflect the share of a given variant in the studied region. For the eight largest (>1%) lineages, the name, number of isolates, and percentage share of total sequences were shown.

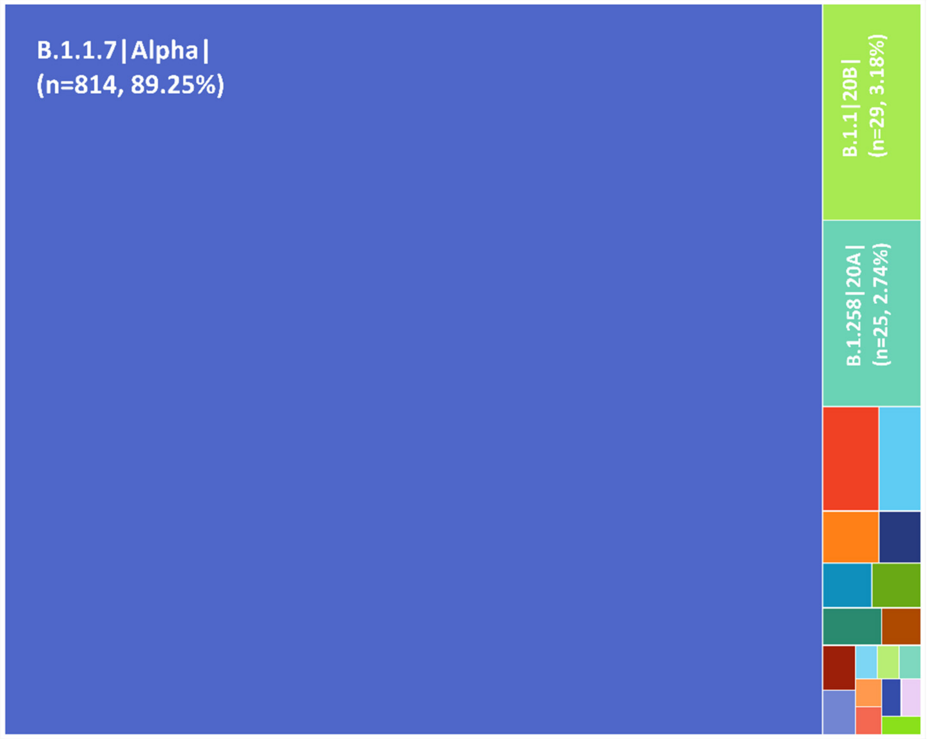

Supplemental Figure S2E. Partition of 912 Lower-Silesia genomes into 21 identified lineages. The size and color of each rectangle reflect the share of a given variant in the studied region. For the three largest (>1%) lineages, the name, number of isolates, and percentage share of total sequences were shown.

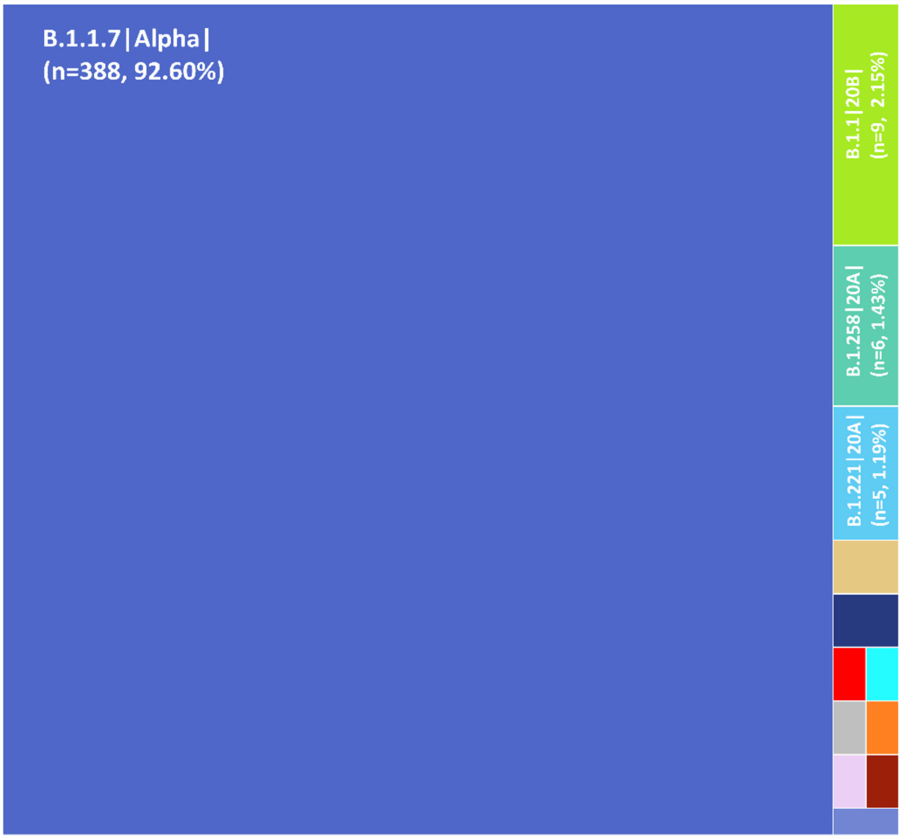

Supplemental Figure S2F. Partition of 419 Lubusz genomes into 13 identified lineages. The size and color of each rectangle reflect the share of a given variant in the studied region. For the four largest (>1%) lineages, the name, number of isolates, and percentage share of total sequences were shown.

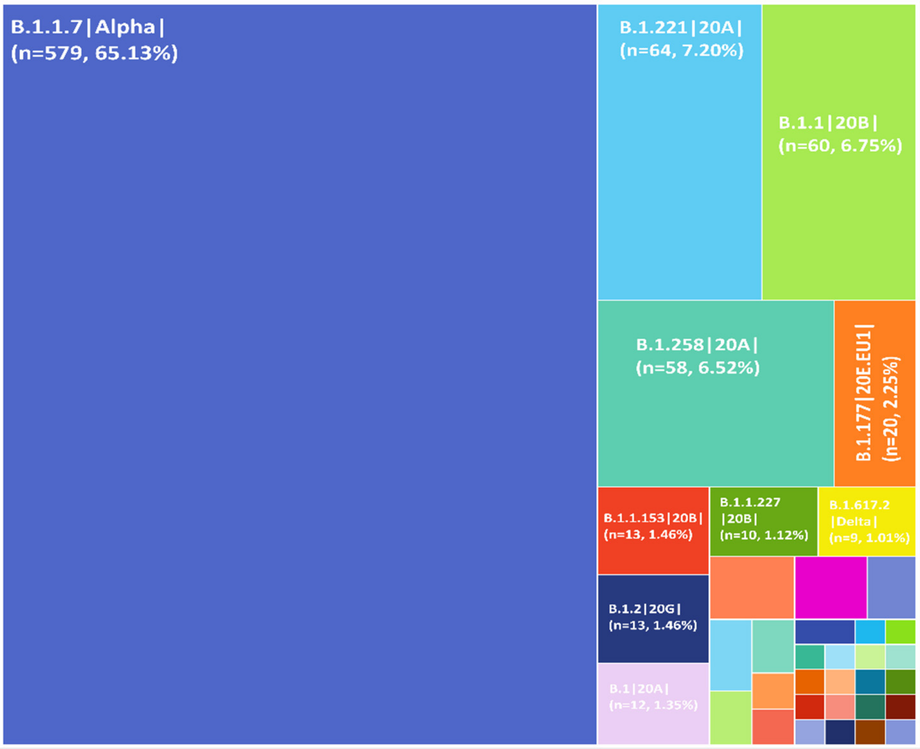

Supplemental Figure S2G. Partition of 889 West-Pomerania genomes into 37 identified lineages. The size and color of each rectangle reflect the share of a given variant in the studied region. For the ten largest (>1%) lineages, the name, number of isolates, and percentage share of total sequences were shown

Lineage size breakdown in state of Berlin showed the distribution of 14 major SARS-CoV-2 lineages circulating between March 2020 and May 2021. During the first two months of analysis, the dominant virus variant was B.1|20A|, with 41 cases observed in March 2020 (75.93%) and 31 (46.97) in April 2020. From May 2020 to August 2020, variant B.1.1|20B| dominated each month: 5 (41.67%), 15 (36.59%), 6 (37.50%), and 8 (21.05%) of sequenced isolates, respectively. In September 2020, lineage B.1.177|20E.EU1| emerged, accounting for 25 (23.81%) cases. In October 2021, the most common virus type was B.1.1.177.86|20E.EU1|, with 17 (17.71%) isolates. In our time trend analysis, samples obtained for November 2020 (12) and December 2020 (3) were excluded due to few sequences. In January 2021, the main virus strain was B.1.1.177.86|20E.EU1| 65 (36.31%). From February 2021 until end of May 2021 variant B.1.1.7|Alpha| showed the highest rate, with 365 (51.97%), 1302 (83.78%), 1156 (88.11%), and 290 (69.38%) cases for each respective month. As for Brandenburg, we revealed ten major SARS-CoV-2 lineages circulating between December 2020 and May 2021. For the time trend analysis related to lineage frequency, sequences obtained before December were excluded due to few samples. During the first month of analysis, the dominant virus lineages were B.1.1.153|20B| and B.1.258|20A|, with 3 (27.27%) cases. In January 2021, isolates with variant B.1.177.86|20E.EU1| peaked, which accounted for 50 (33.56%) sequences. From February 2021 until end of May 2021, variant B.1.1.7|Alpha| showed the highest rate, with 148 (35.83%), 261 (77.81%), 291 (94.48%), and 99 (89.19%) cases for each respective month. In Mecklenburg-West-Pomerania occurred, seven major SARS-CoV-2 lineages circulated between January and May 2021. For the time trend analysis related to lineage frequency, sequences obtained before January were excluded due to few samples. During the first month of analysis, the dominant virus lineage was B.1.177|20E.EU1|, with 31 (69.37%) cases. From February 2021 until the end of May 2021, variant B.1.1.7|Alpha| showed the highest rate, with 76 (53.15%), 118 (72.83%), 748 (92.00%), and 94 (98.95%) cases for each respective month. Saxony sequences displayed patterns of eight major SARS-CoV-2 lineages circulating between December 2020 and May 2021. For the time trend analysis related to lineage frequency, sequences obtained before December were excluded due to few samples. During the first month of analysis, the dominant virus lineage was B.1.258|20A|, with 15 (55.56%) cases. In January 2021, variant B.1.160|20A.EU2| caused 173 (16.18%) cases. From February 2021 until the end of May 2021, lineage B.1.1.7|Alpha| showed the highest rate, with 826 (37.89%), 2532 (79.82%), 2730 (95.19%), and 955 (94.65%) cases for each respective month. In Lower-Silesia, we unveiled nine major SARS-CoV-2 lineages circulating between December 2020 and May 2021. For the time trend analysis related to lineage frequency, sequences obtained before December were excluded due to few samples. During the first month of analysis, the dominant virus lineages were B.1.1.153|20B| and B.1.1|20B|, both with four (30.77%) cases. In January 2021, only four sequences were observed, with a single case for each of B.1.1.7|Alpha|, B.1.258|20A|, B.1.221|20A|, and B.1.177|20E.EU1|. From February 2021 until the end of May 2021, lineage B.1.1.7|Alpha| showed the highest rate, with 103 (69.13%), 263 (91.32%), 361 (98.63%), and 86 (96.63%) cases for each respective month. In Lubusz we showed eight major SARS-CoV-2 lineages circulating between December 2020 and May 2021. For the time trend analysis related to lineage frequency, sequences obtained before December were excluded due to few samples. During the first month of analysis, the dominant virus lineage was B.1.221|20A|, with 4 (66.67%) cases. In January 2021, a single

case was observed for both B.1.2|20G| and B.1.258|20A|. From February 2021 until the end of May 2021, lineage B.1.1.7|Alpha| showed the highest contribution, with 9 (64.29%), 139 (95.21%), 175 (98.87%), and 65 (89.04%) cases each month, respectively. In West-Pomerania, we showed nine major SARS-CoV-2 lineages circulating between November 2020 and May 2021. For the time trend analysis related to lineage frequency, sequences obtained before November were excluded due to few samples. During the first month of analysis, the dominant virus lineages were B.1.1|20B| and B.1.221|20A|, both with two (40.00%) cases. In December 2020, the most prevalent variant was B.1.258|20A|, with 32 (32.00%) genomes. In January 2021, 27 (33.33%) B.1.221|20A| cases were recorded as the highest virus variant prevalence. From February 2021 until the end of May 2021, B.1.1.7|Alpha| showed the highest rate, with 35 (35.35%), 197 (82.43%), 314 (98.13%), and 31 (79.49%) cases for each respective month.

Supplemental Figure 3A–E

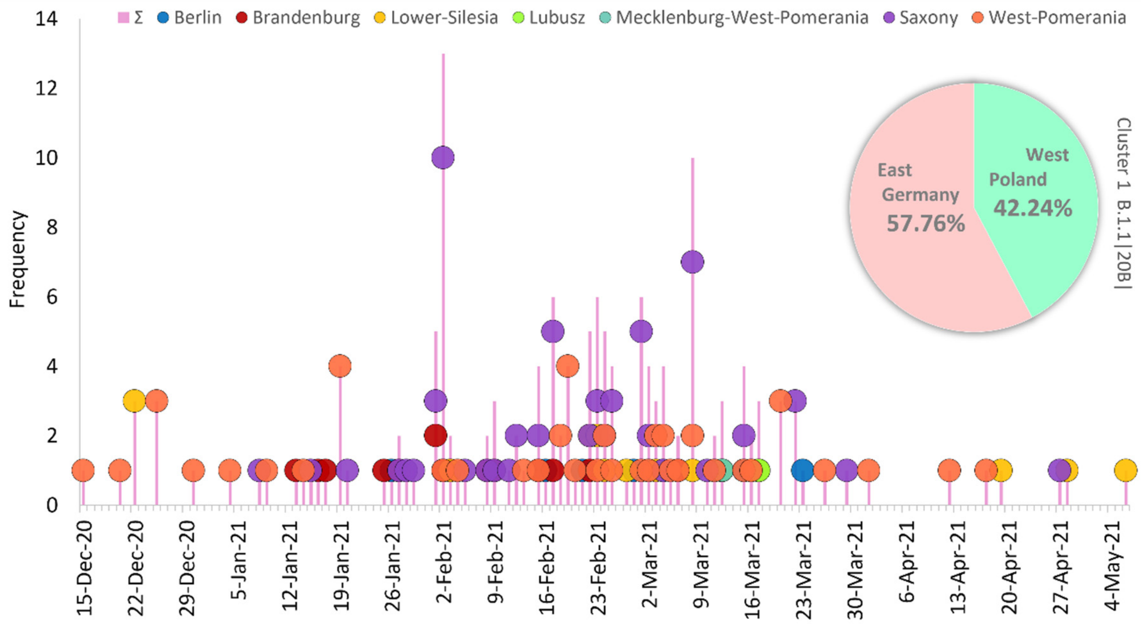

Supplemental Figure S3A. Frequency and distribution of Cluster 1 (B.1.1|20B|) circulating in Polish-German borderland area over time. The pie chart shows the percentage of sequences identified for a given country. The cluster was comprised of 161 sequences, 93 from East Germany and 68 from West Poland. The first isolate was detected on 15-Dec-2020 in West Pomerania, and the last sample was noted on 6-May-2021 in Lower-Silesia.

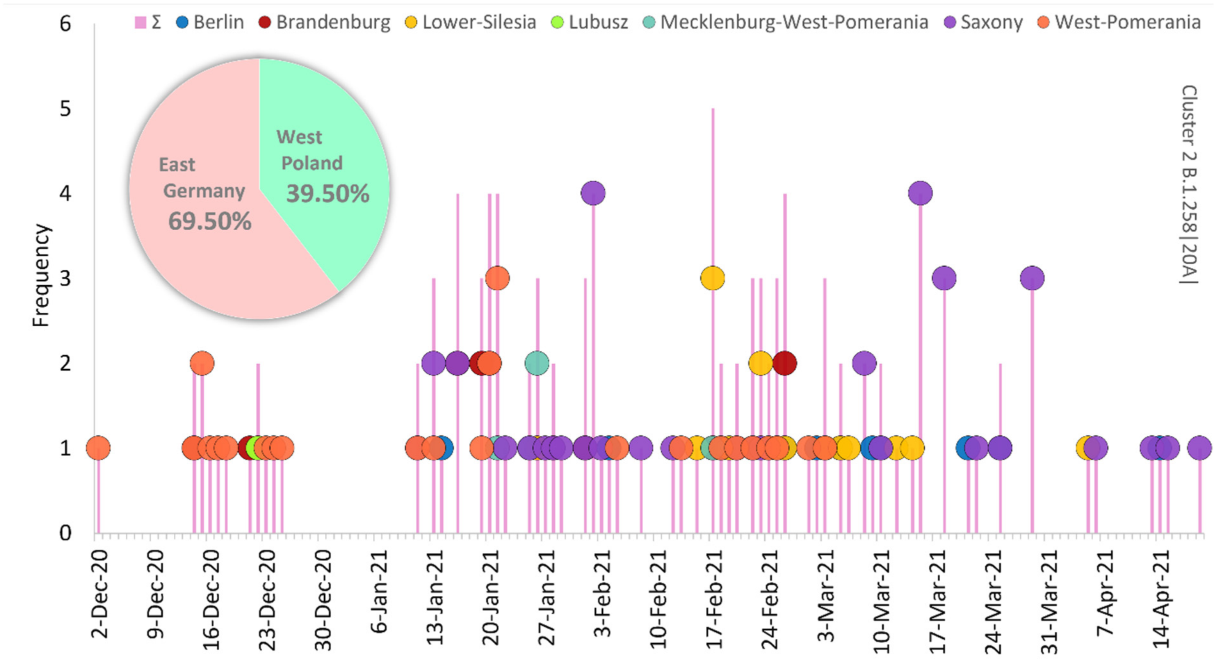

Supplemental Figure S3B. Frequency and distribution of Cluster 2 (B.1.258|20A|) circulating in Polish-German borderland area over time. The pie chart shows the percentage of sequences identified for a given country. The cluster was comprised of 119 sequences, 72 from East Germany and 47 from West Poland. The first isolate was detected on 2-Dec-2020 in West Pomerania, and the last sample was noted on 19-Apr-2021 in Saxony.

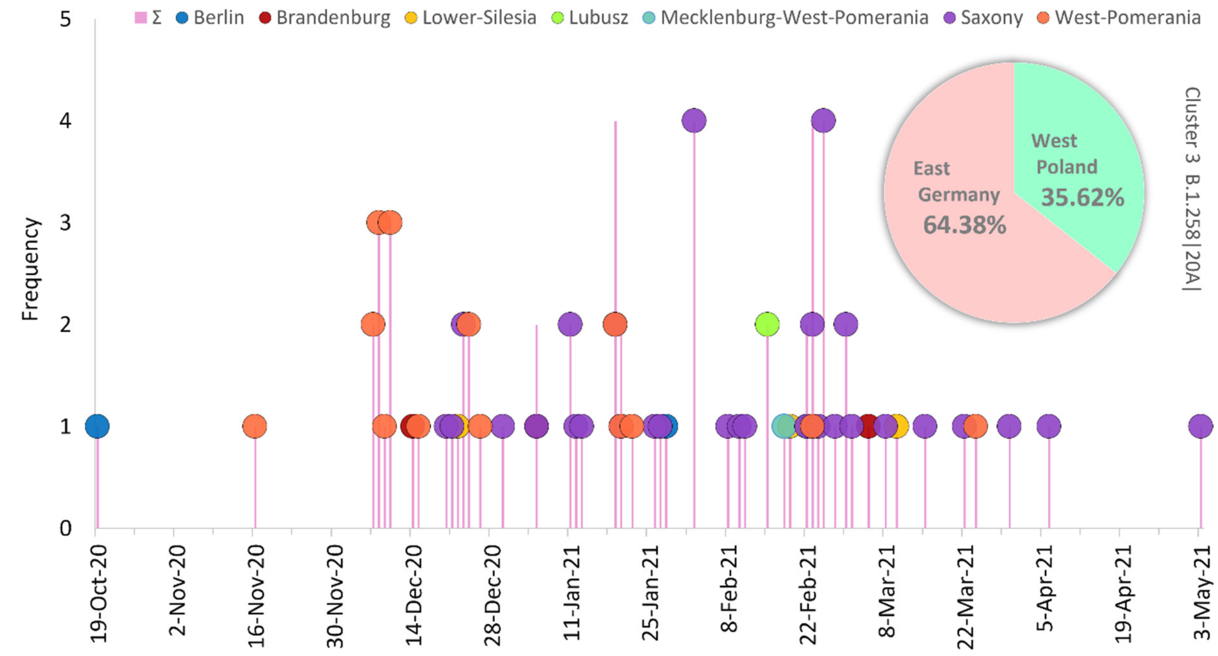

Supplemental Figure S3C. Frequency and distribution of Cluster 3 (B.1.258|20A|) circulating in Polish-German borderland area over time. The pie chart shows the percentage of sequences identified for a given country. The cluster was comprised of 73 sequences, 47 from East Germany and 26 from West Poland. The first isolate was detected on 19-Oct-2020 in Berlin, and the last sample was noted on 3-May-2021 in Saxony.

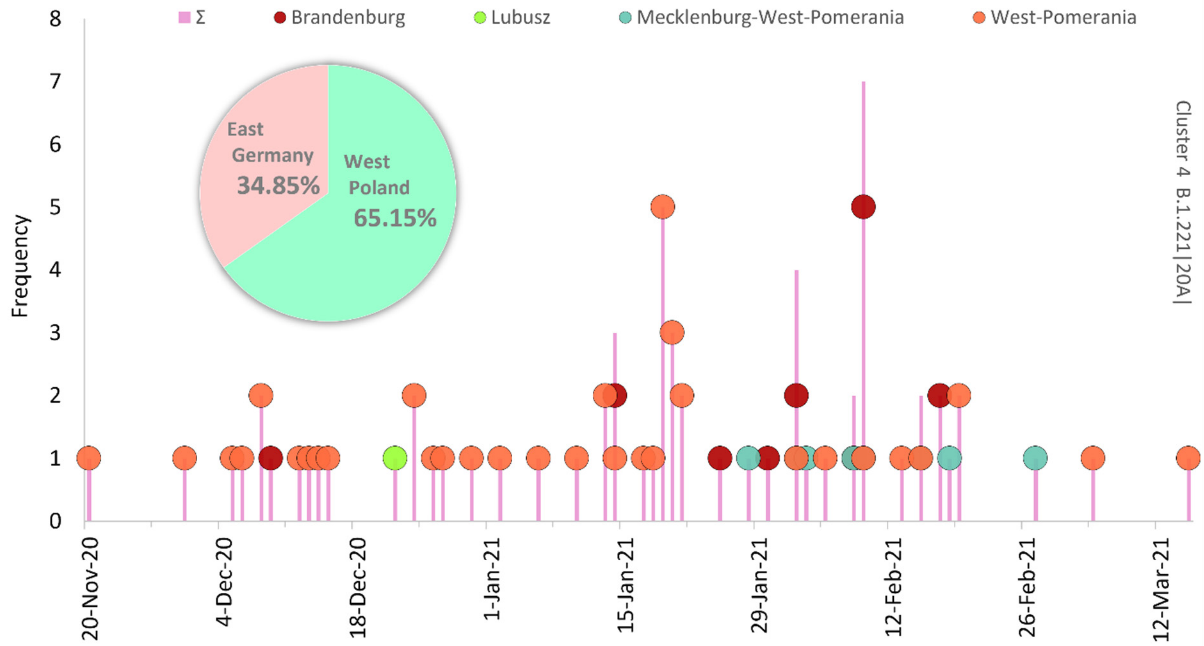

Supplemental Figure S3D. Frequency and distribution of Cluster 4 (B.1.221|20A|) circulating in Polish-German borderland area over time. The pie chart shows the percentage of sequences identified for a given country. The cluster was comprised of 66 sequences, 23 from East Germany and 43 from West Poland. The first isolate was detected on 20-Nov-2020 in West-Pomerania, and the last sample was noted on 15-Mar-2021 also in West-Pomerania.

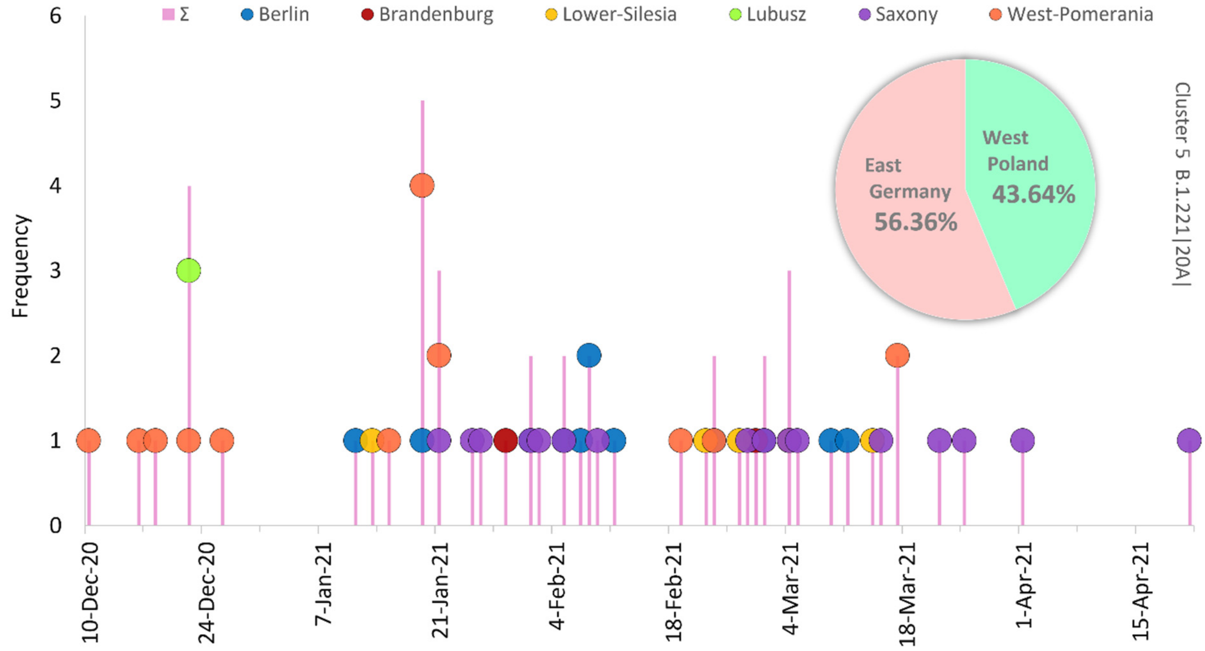

Supplemental Figure S3E. Frequency and distribution of Cluster 5 (B.1.221|20A|) circulating in Polish-German borderland area over time. The pie chart shows the percentage of sequences identified for a given country. The cluster was comprised of 55 sequences, 31 from East Germany and 24 from West Poland. The first isolate was detected on 10-Dec-2020 in West-Pomerania, and the last sample was noted on 21-Apr-2021 in Saxony.

Supplemental Figure S4A–E

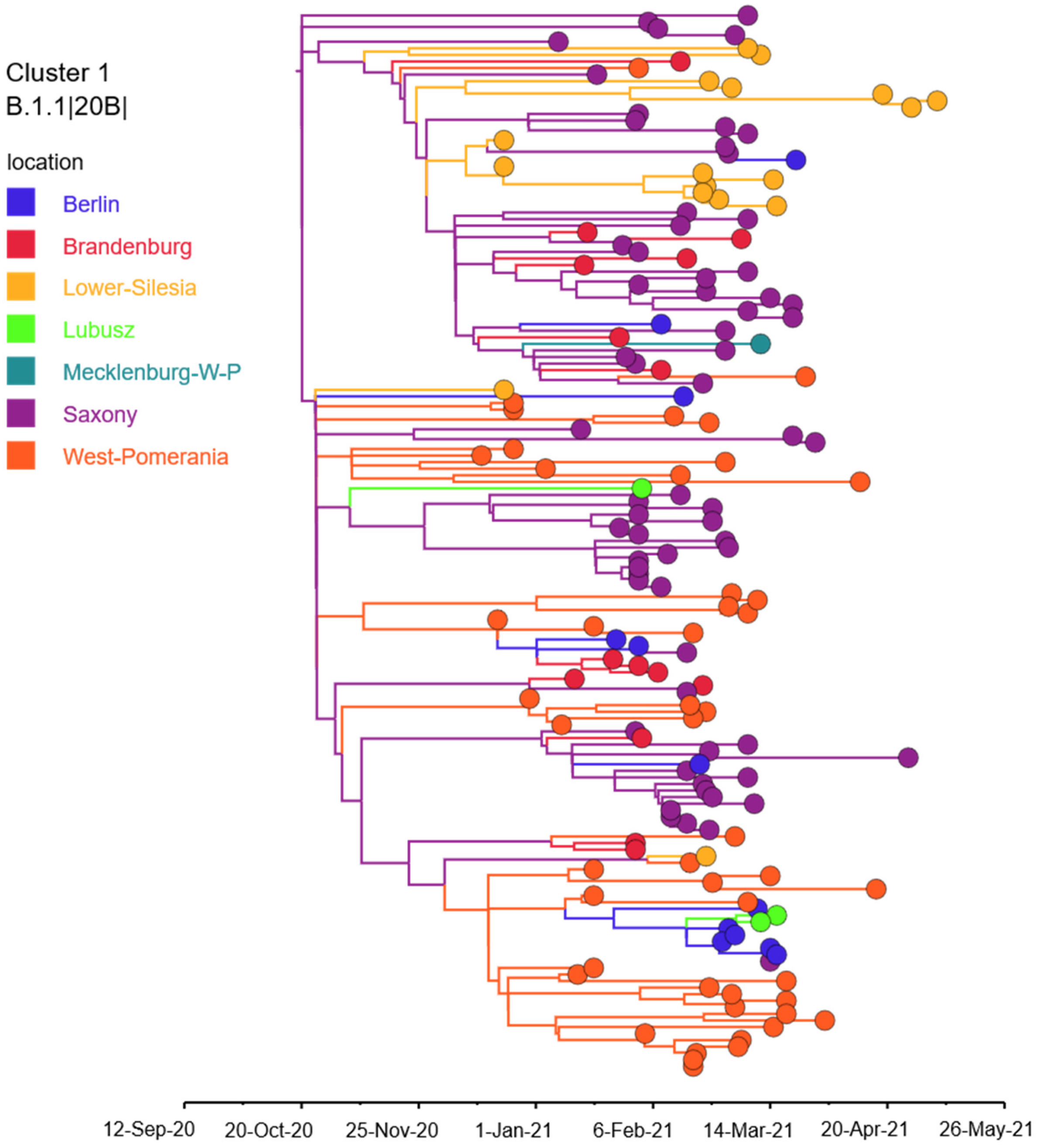

Supplemental Figure S4A. Time-resolved maximum clade credibility phylogeny of the Cluster 1 (B.1.1|20B|) among Polish-German borderland area, with province location indicated. Time to the most recent common ancestor (TMRCA) of clade was estimated on 20-Oct-2020.

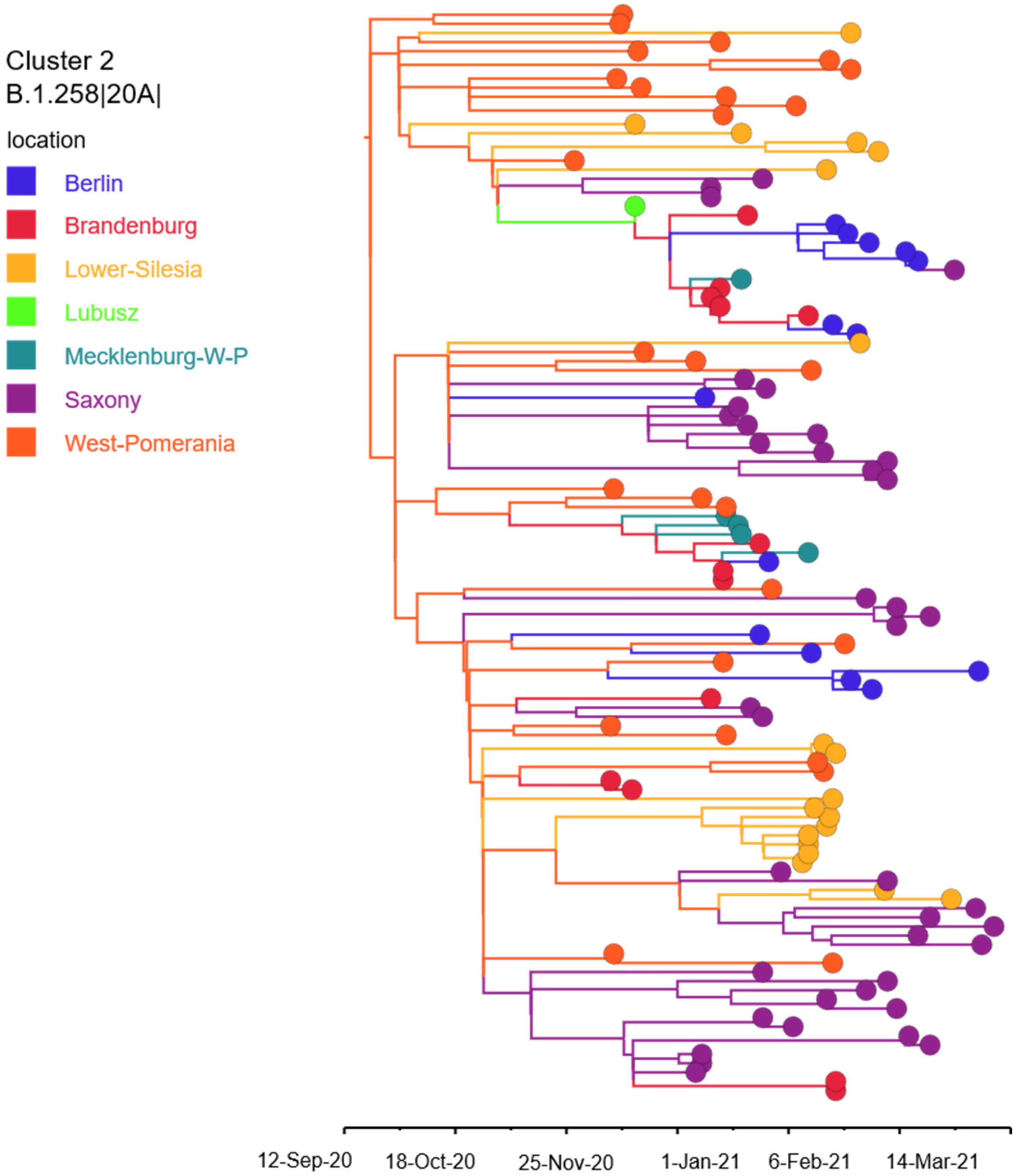

Supplemental Figure S4B. Time-resolved maximum clade credibility phylogeny of the Cluster 2 (B.1.258|20A|) among Polish-German borderland area, with province location indicated. Time to the most recent common ancestor (TMRCA) of clade was estimated on 26-Sep-2020.

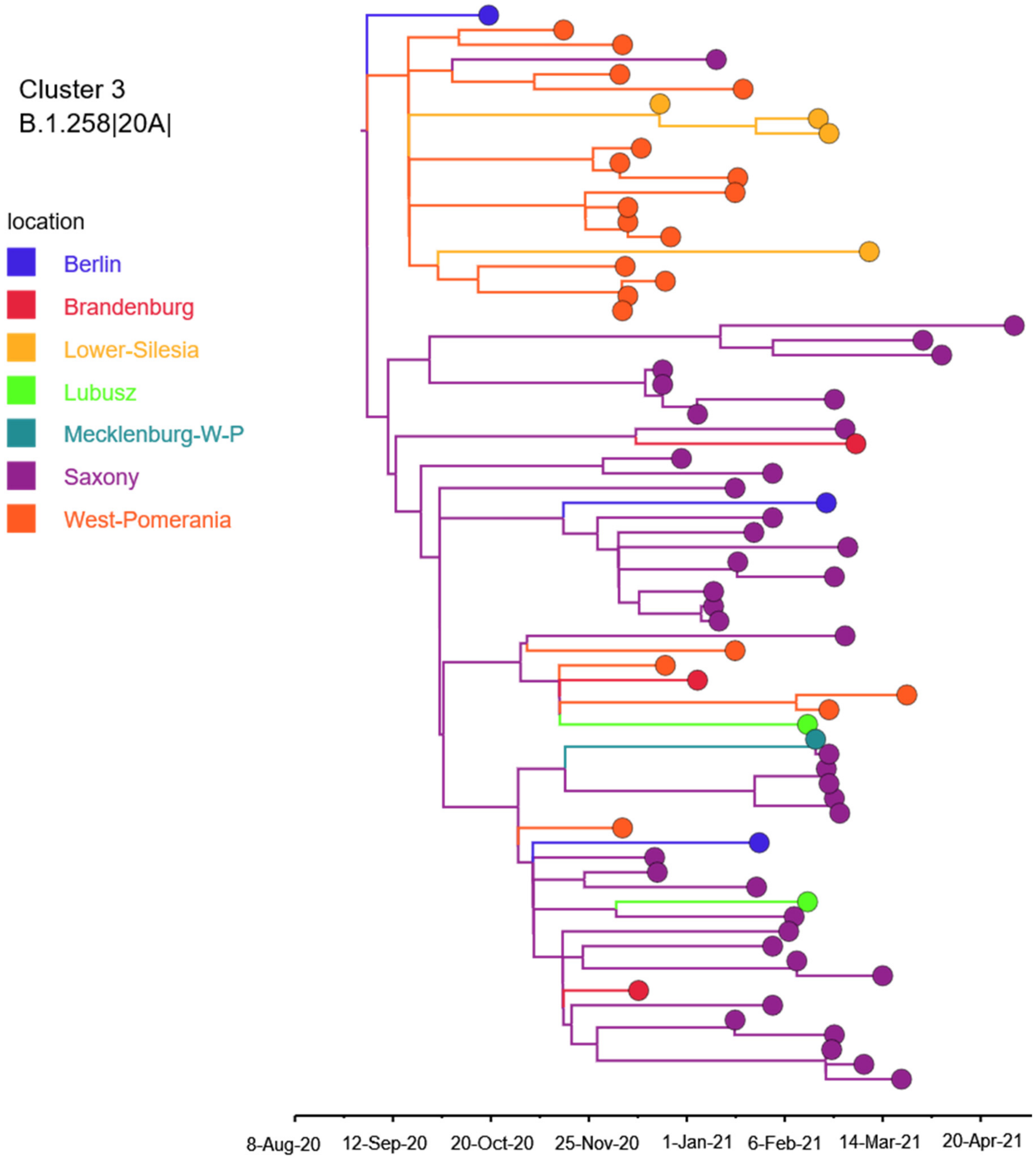

Supplemental Figure S4C. Time-resolved maximum clade credibility phylogeny of the Cluster 3 (B.1.258|20A|) among Polish-German borderland area, with province location indicated. Time to the most recent common ancestor (TMRCA) of clade was estimated on 4-Sep-2020.

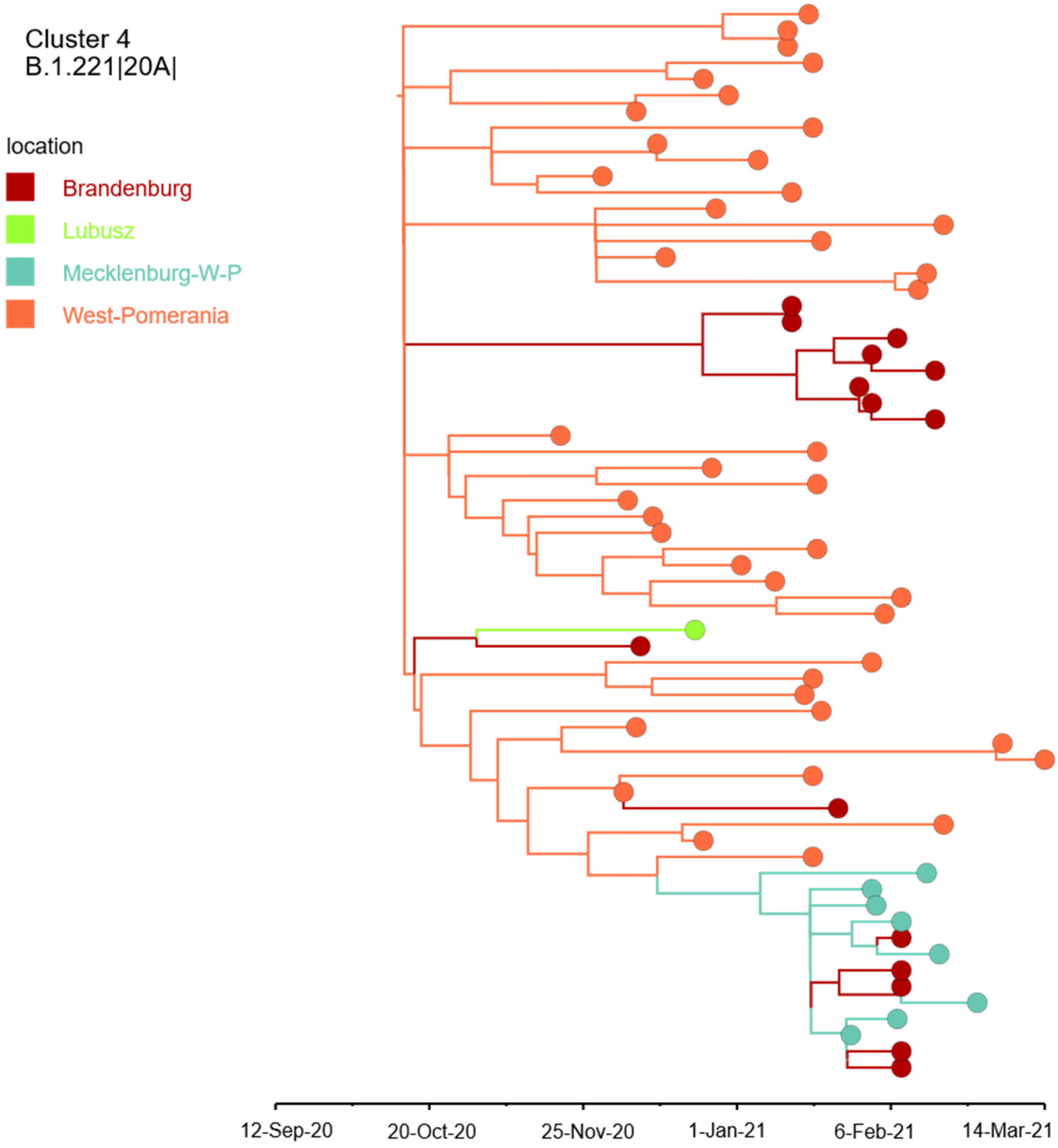

Supplemental Figure S4D. Time-resolved maximum clade credibility phylogeny of the Cluster 3 (B.1.258|20A|) among Polish-German borderland area, with province location indicated. Time to the most recent common ancestor (TMRCA) of clade was estimated on 14-Oct-2020.

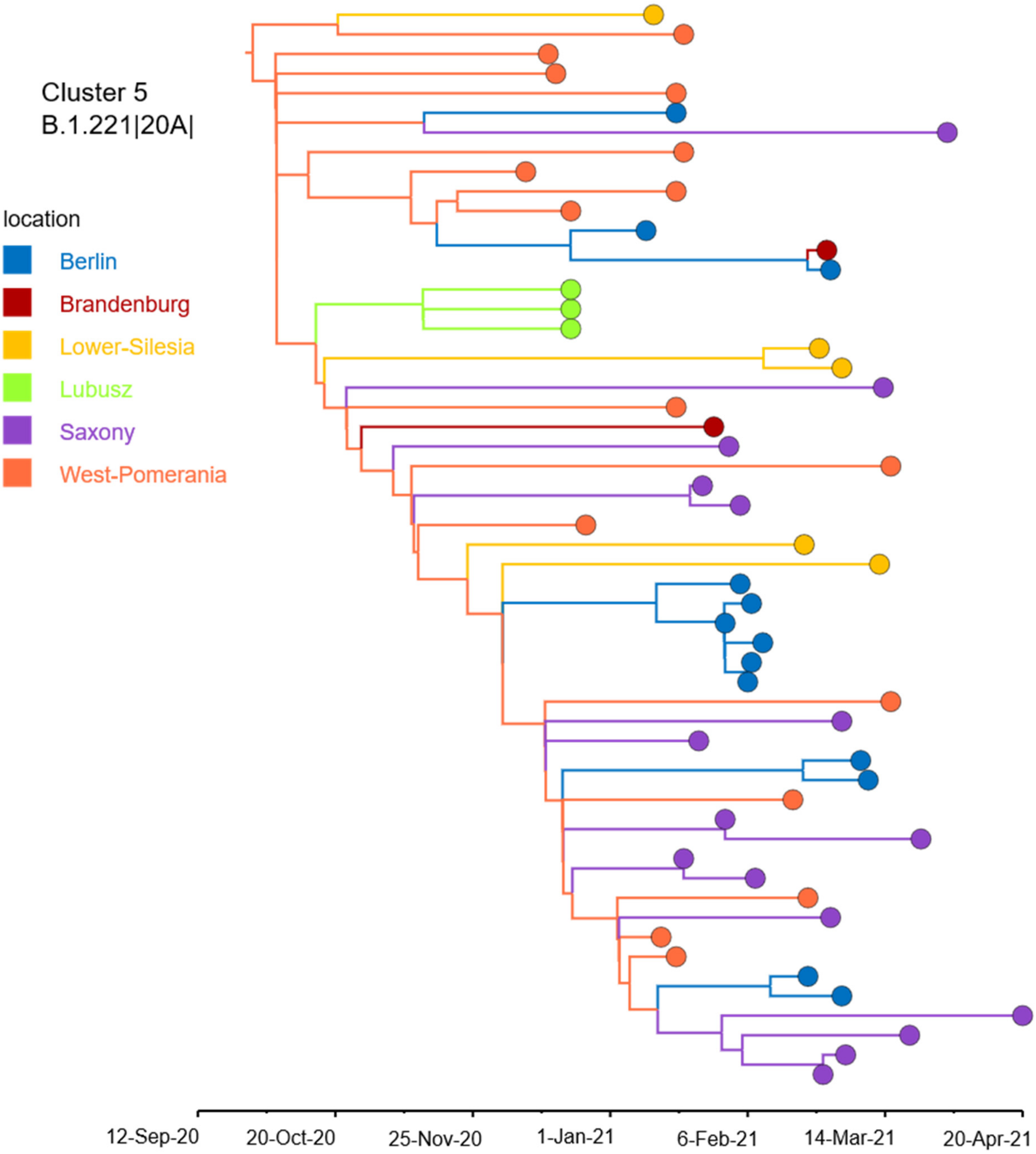

Supplemental Figure S4E. Time-resolved maximum clade credibility phylogeny of the Cluster 3 (B.1.258|20A|) among Polish-German borderland area, with province location indicated. Time to the most recent common ancestor (TMRCA) of clade was estimated on 27-Sep-2020.

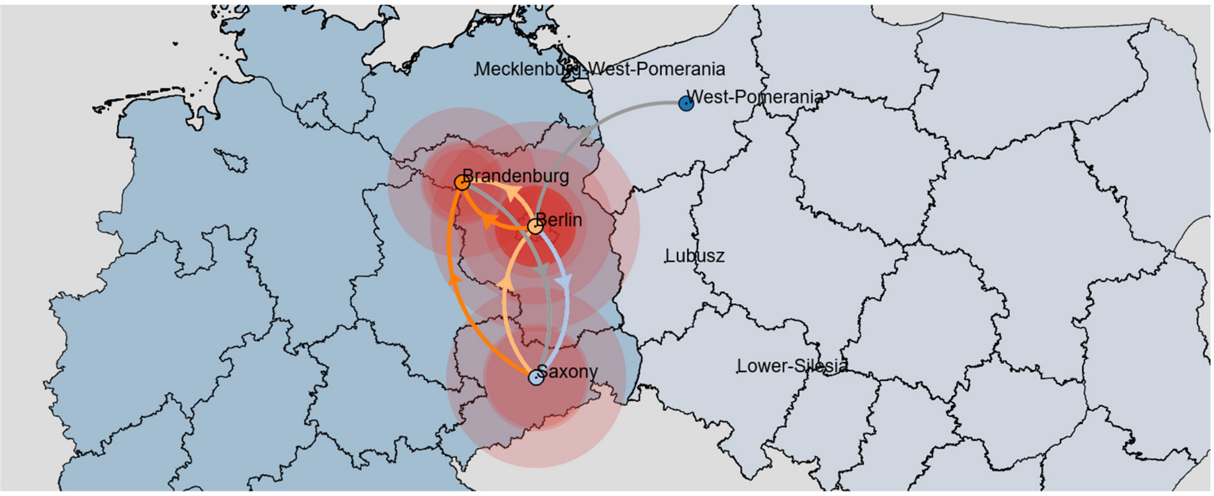

Supplemental Figure S5. Reconstruction of the dispersal history of the largest identified non-Alpha Cluster among Polish-German borderline area under discrete diffusion model. Each of the seven regions was indicated by a (centroid) circle with different colors, and inward movements to a particular location were depicted in the same color with an arrow. The size of the polygons around the sampling centers was proportional to the number of viruses that maintain a specific area. All migration events between locations were supported by Bayes Factor values ≥ 3, except lines indicated in grey color. Base layer for the maps has been obtained from Stanford Digital Repository.

Supplemental Figure S6 A–G

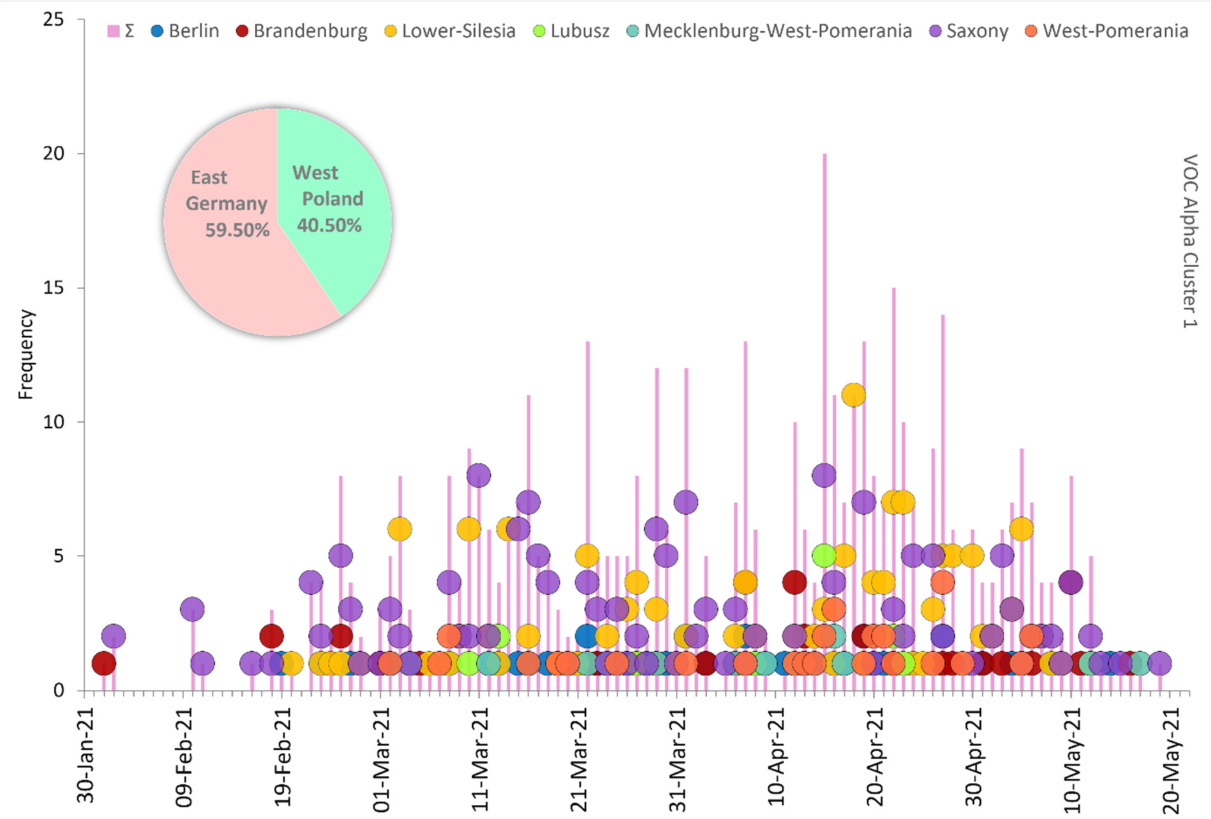

Supplemental Figure S6A. Frequency and distribution of the VOC Alpha Cluster 1 circulating in Polish-German borderland area over time. The pie chart shows the percentage of sequences identified for a given country. The cluster was comprised of 484 sequences, 288 from East Germany and 196 from West Poland. The first isolate was detected on 1-Feb-2021 in Saxony, and the last sample was noted on 19-May-2021 in Saxony.

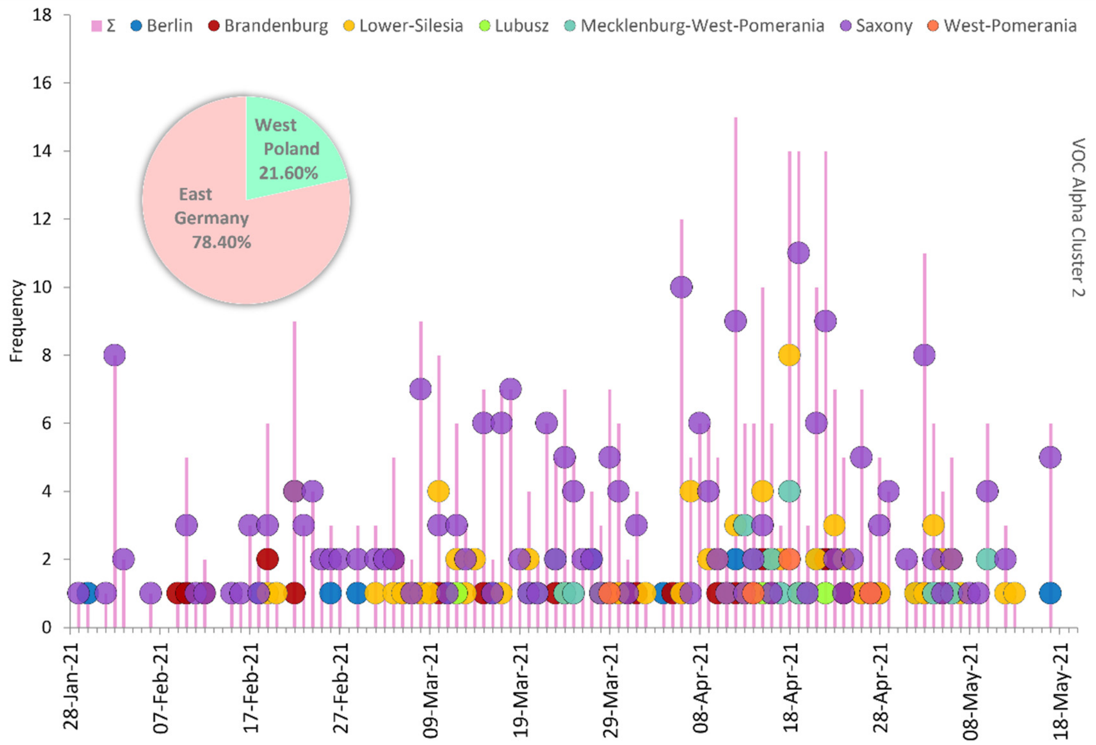

Supplemental Figure S6B. Frequency and distribution of the VOC Alpha Cluster 2 circulating in Polish-German borderland area over time. The pie chart shows the percentage of sequences identified for a given country. The cluster was comprised of 323 sequences, 95 from East Germany and 89 from West Poland. The first isolate was detected on 29-Jan-2021 in Saxony, and the last sample was noted on 17-May-2021 in Berlin and Brandenburg.

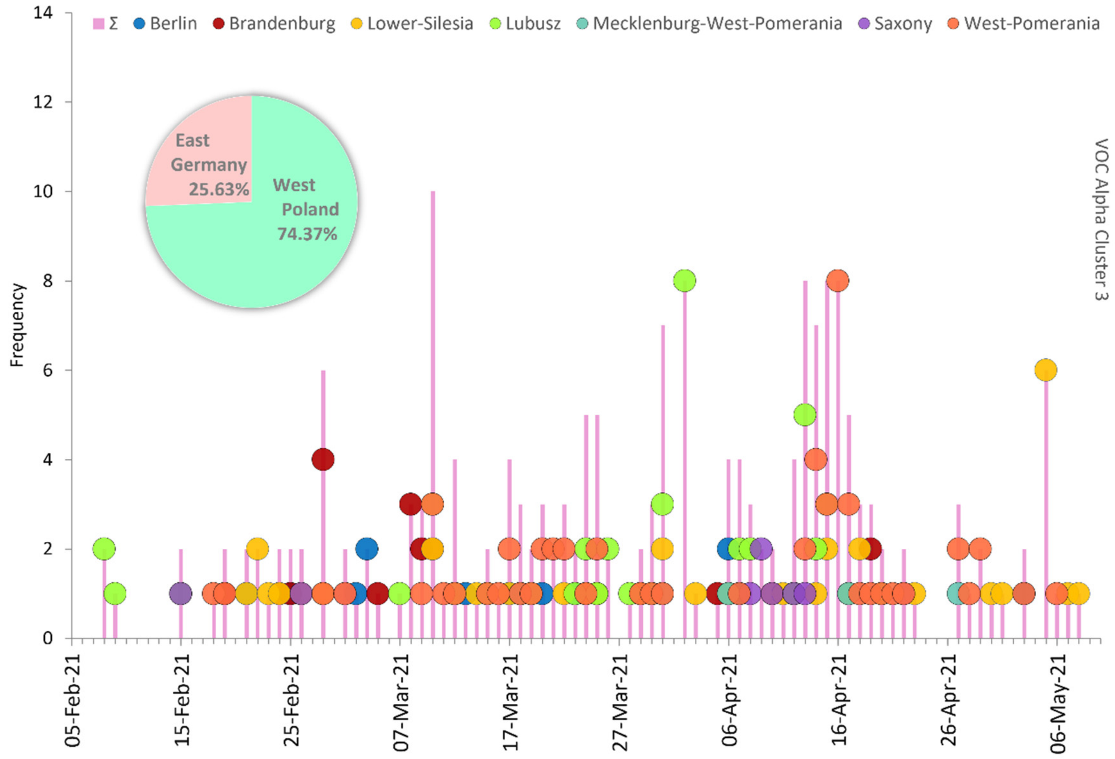

Supplemental Figure S6C. Frequency and distribution of the VOC Alpha Cluster 3 circulating in Polish-German borderland area over time. The pie chart shows the percentage of sequences identified for a given country. The cluster was comprised of 199 sequences, 51 from East Germany and 148 from West Poland. The first isolate was detected on 8-Feb-2021 in Lubusz, and the last sample was noted on 8-May-2021 also in Lower Silesia.

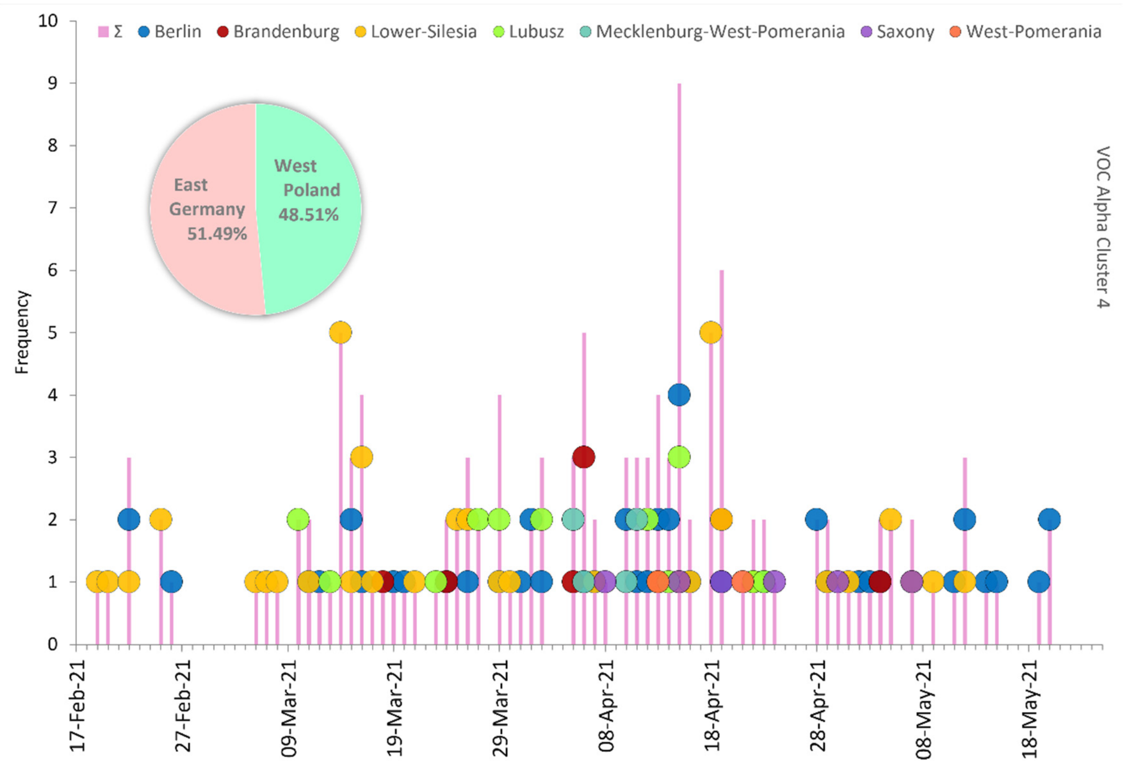

Supplemental Figure S6D. Frequency and distribution of the VOC Alpha Cluster 4 circulating in Polish-German borderland area over time. The pie chart shows the percentage of sequences identified for a given country. The cluster was comprised of 134 sequences, 69 from East Germany and 65 from West Poland. The first isolate was detected on 19-Feb-2021 in Lower-Silesia, and the last sample was noted on 20-May-2021 also in Berlin.

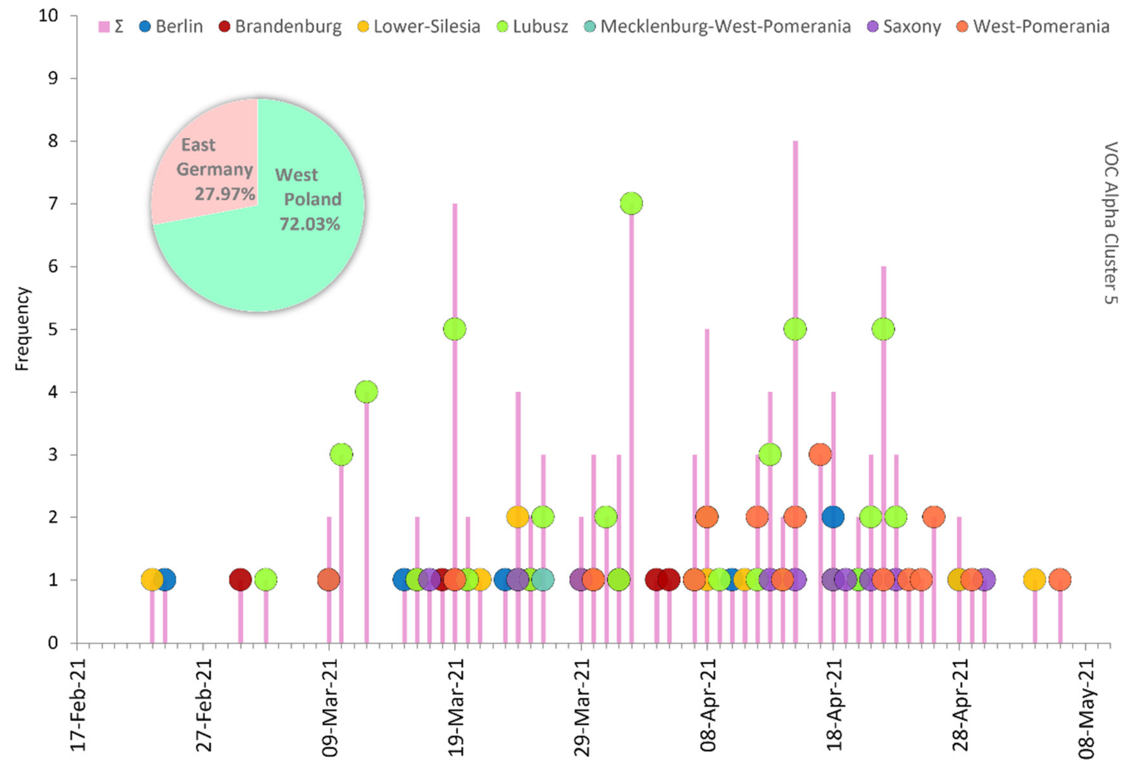

Supplemental Figure S6E. Frequency and distribution of the VOC Alpha Cluster 5 circulating in Polish-German borderland area over time. The pie chart shows the percentage of sequences identified for a given country. The cluster was comprised of 118 sequences, 33 from East Germany and 85 from West Poland. The first isolate was detected on 23-Feb-2021 in Lower Silesia, and the last sample was noted on 12-May-2021 in West Pomerania.

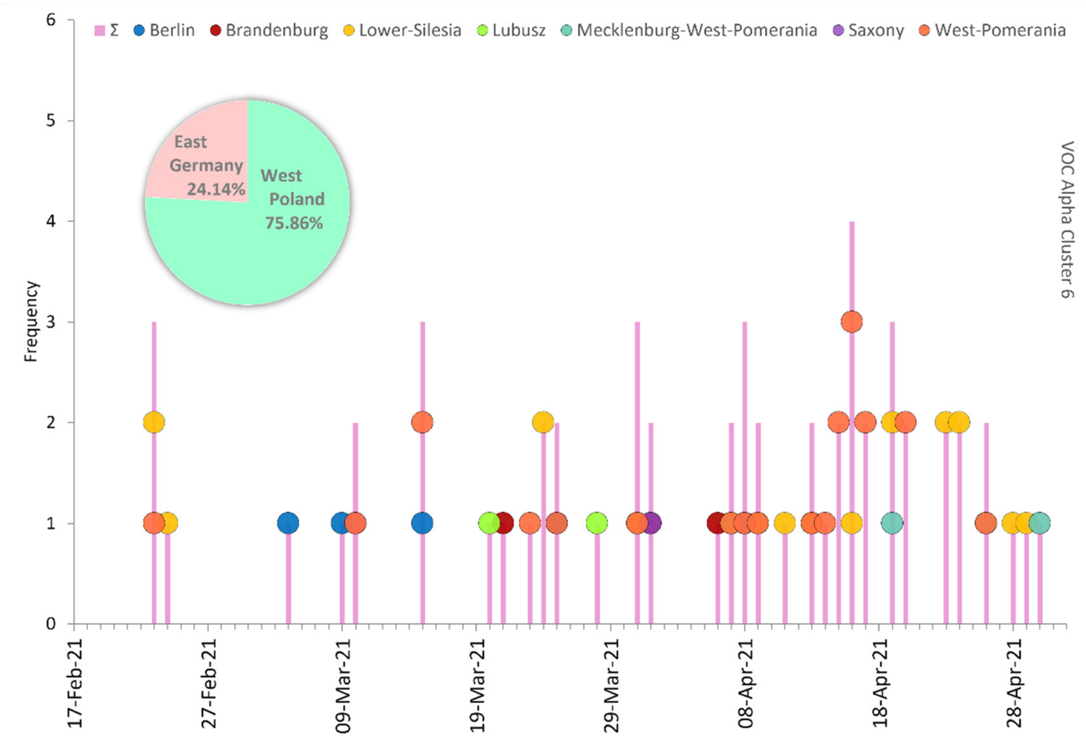

Supplemental Figure S6F. Frequency and distribution of the VOC Alpha Cluster 6 circulating in Polish-German borderland area over time. The pie chart shows the percentage of sequences identified for a given country. The cluster was comprised of 58 sequences, 14 from East Germany and 44 from West Poland. The first isolate was detected on 23-Feb-2021 in Lower Silesia and West Pomerania. The last sample was noted on 30-Apr-2021 in Mecklenburg West Pomerania.

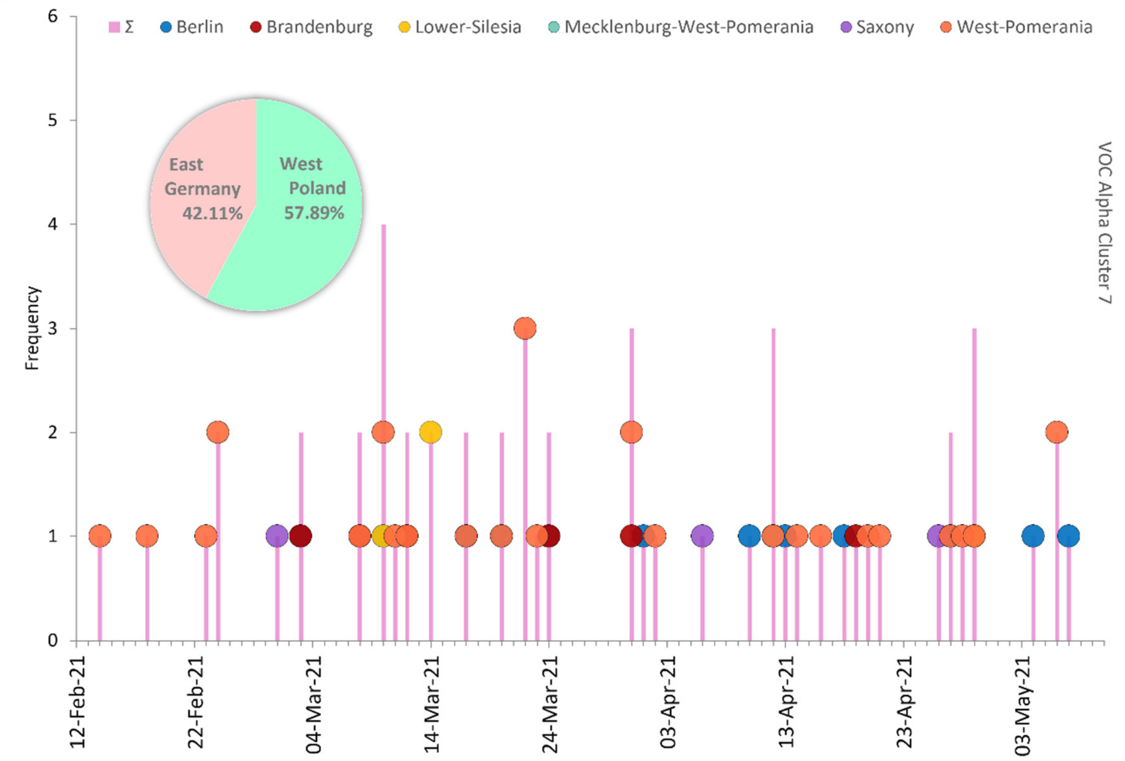

Supplemental Figure S6G. Frequency and distribution of the VOC Alpha Cluster 7 circulating in Polish-German borderland area over time. The pie chart shows the percentage of sequences identified for a given country. The cluster was comprised of 57 sequences, 24 from East Germany and 33 from West Poland. The first isolate was detected on 14-Feb-2021 in West-Pomerania and the last sample was noted on 7-May-2021 in Berlin.

Supplemental Figure 7A–G

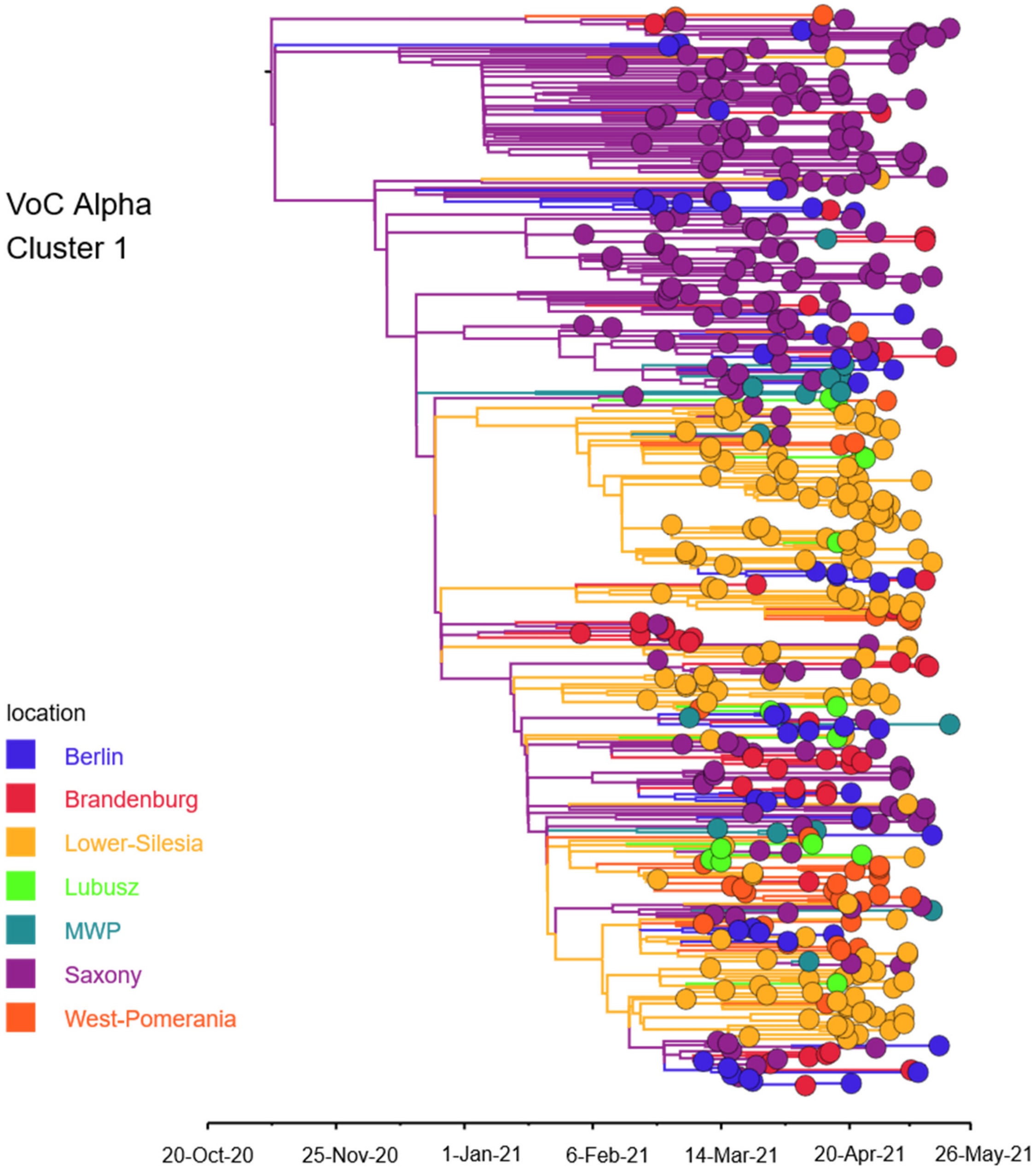

Supplemental Figure S7A. Time-resolved maximum clade credibility phylogeny of the VOC Alpha Cluster 1 among Polish-German borderland area, with province location indicated. Time to the most recent common ancestor (TMRCA) of cluster was estimated on 5-Nov-2020.

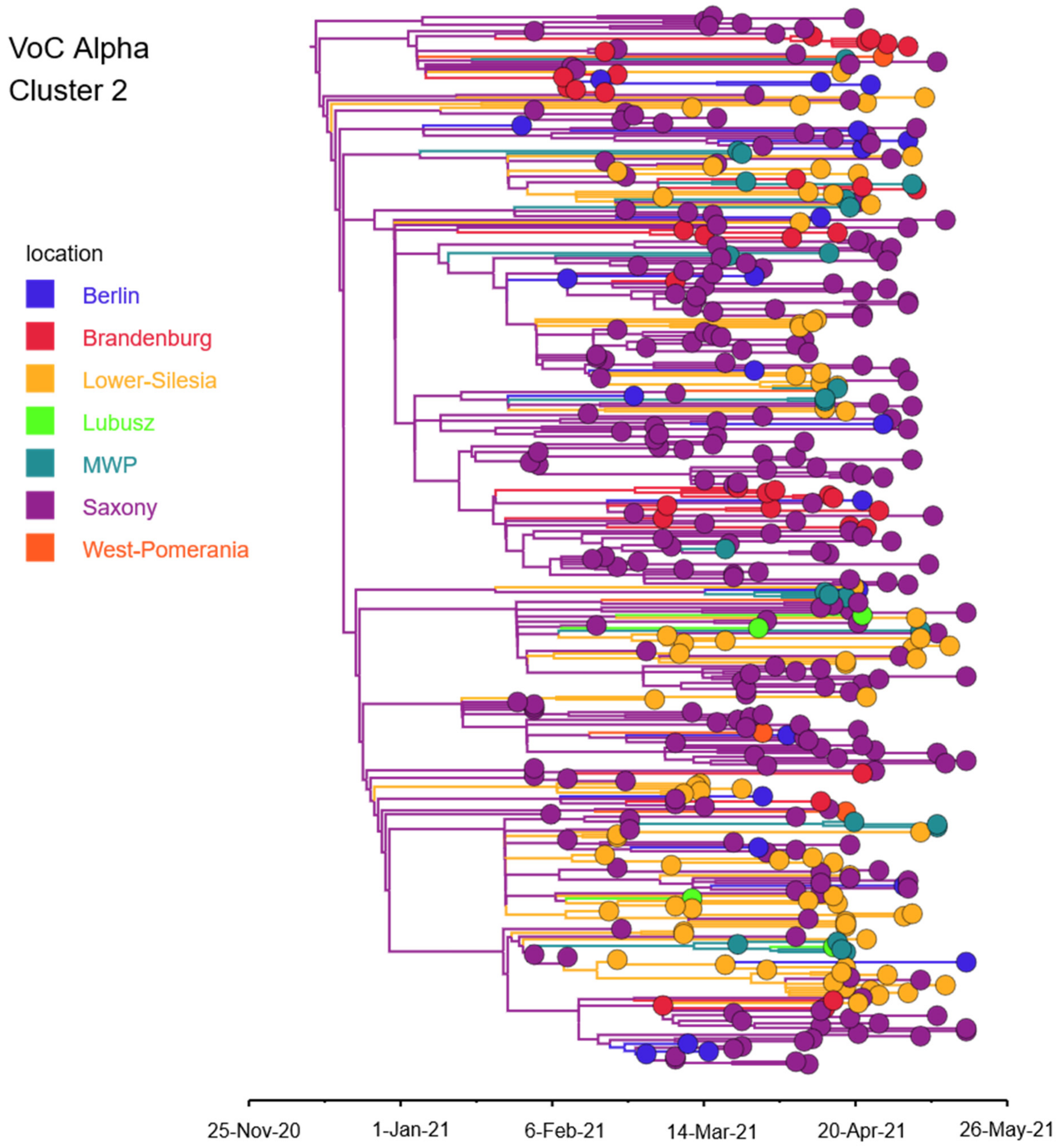

Supplemental Figure S7B. Time-resolved maximum clade credibility phylogeny of the VOC Alpha Cluster 2 among Polish-German borderland area, with province location indicated. Time to the most recent common ancestor (TMRCA) of cluster was estimated on 10-Dec-2020.

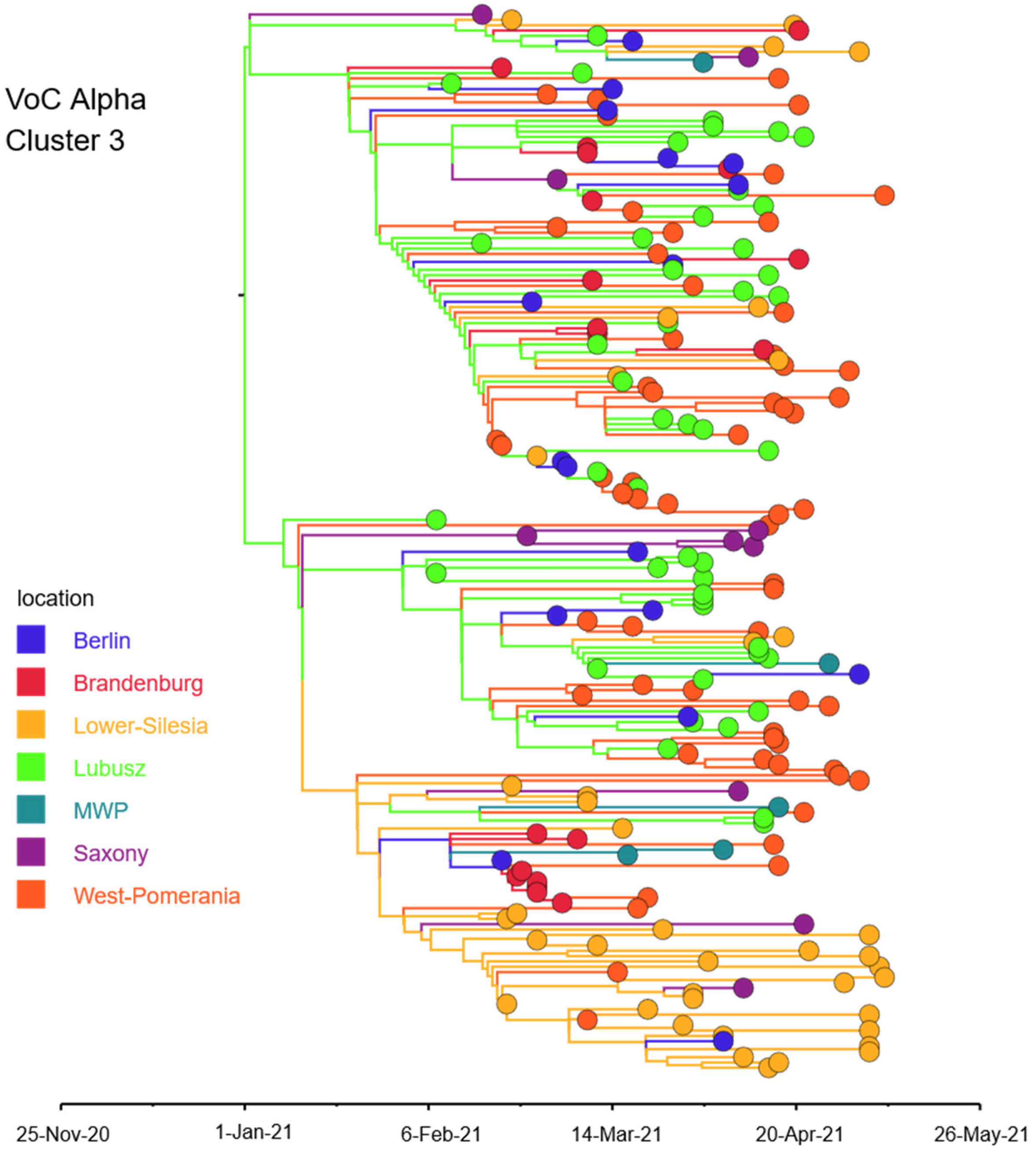

Supplemental Figure S7C. Time-resolved maximum clade credibility phylogeny of the VOC Alpha Cluster 3 among Polish-German borderland area, with province location indicated. Time to the most recent common ancestor (TMRCA) of cluster was estimated on 30-Dec-2020.

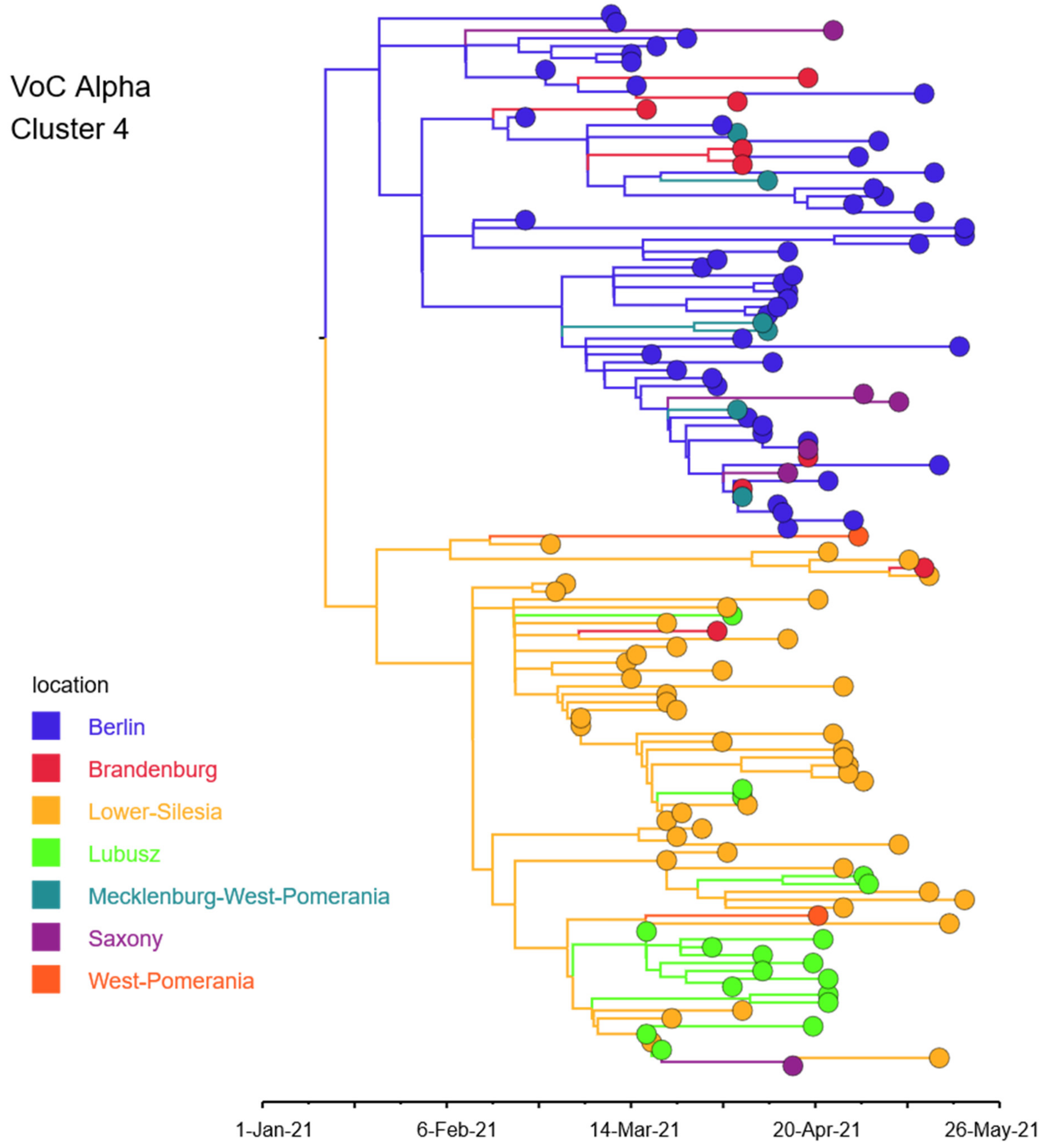

Supplemental Figure S7D. Time-resolved maximum clade credibility phylogeny of the VOC Alpha Cluster 4 among Polish-German borderland area, with province location indicated. Time to the most recent common ancestor (TMRCA) of cluster was estimated on 8-Jan-2021.

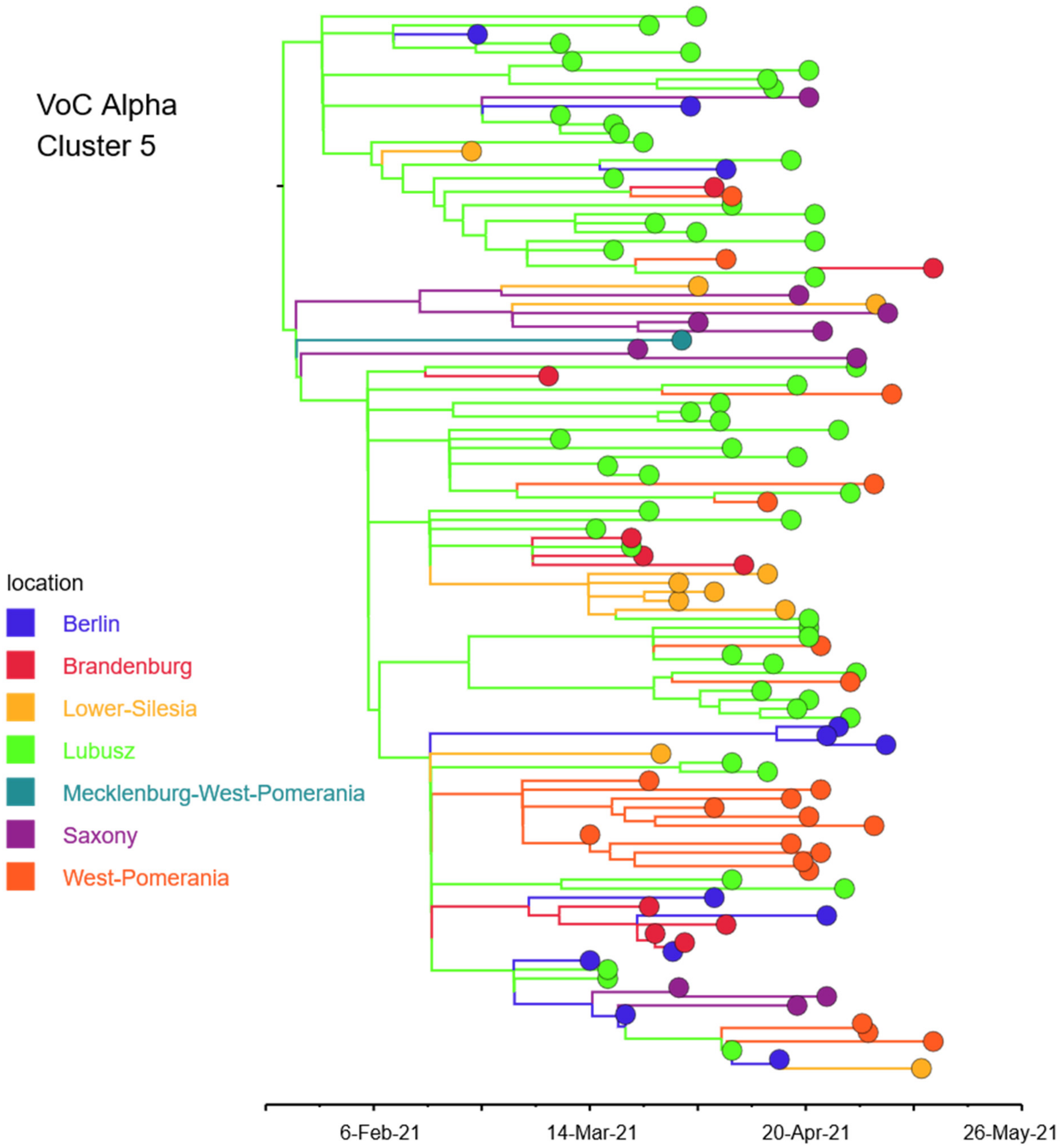

Supplemental Figure S7E. Time-resolved maximum clade credibility phylogeny of the VOC Alpha Cluster 5 among Polish-German borderland area, with province location indicated. Time to the most recent common ancestor (TMRCA) of cluster was estimated on 18-Jan-2021.

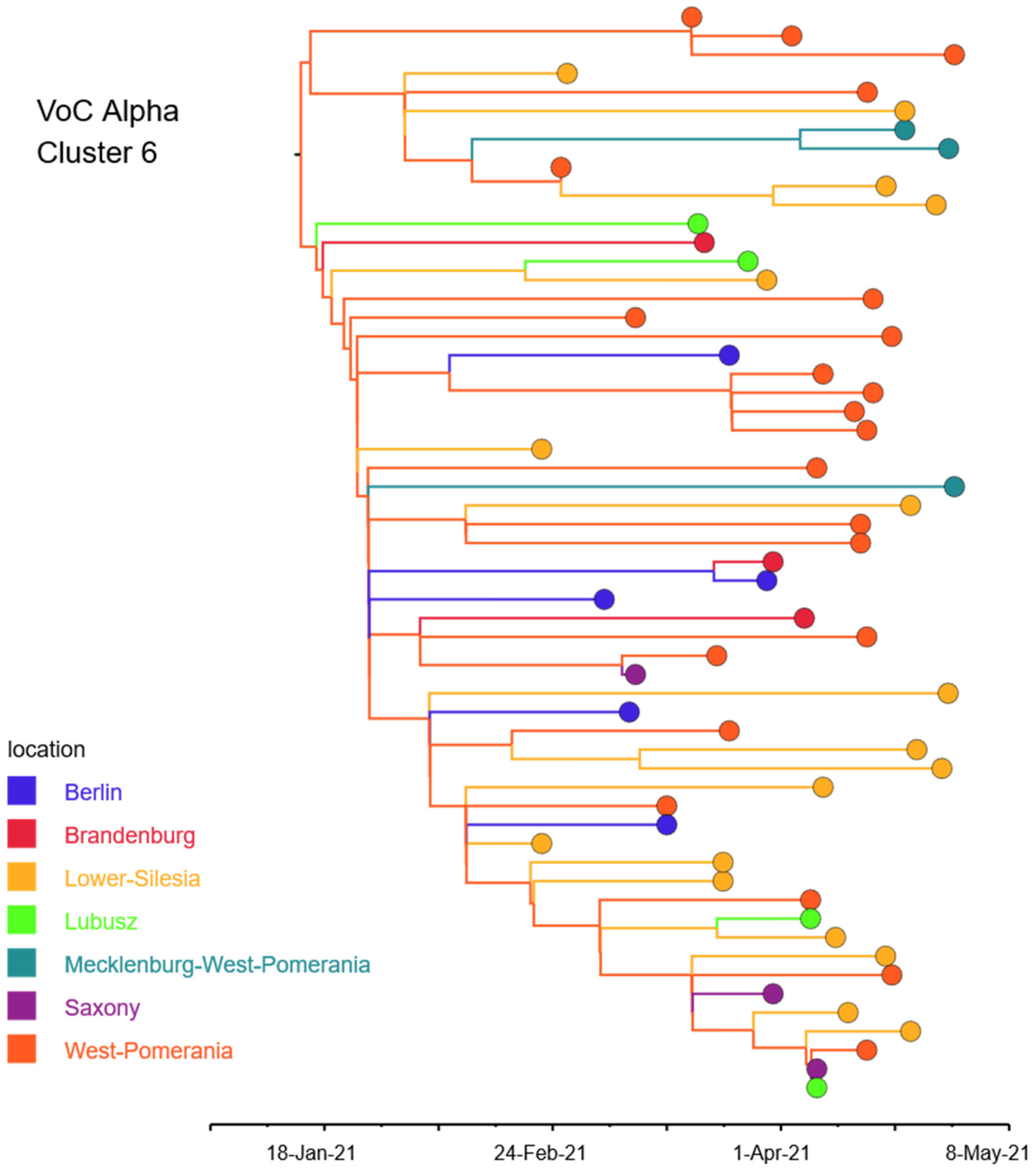

Supplemental Figure S7F. Time-resolved maximum clade credibility phylogeny of the VOC Alpha Cluster 6 among Polish-German borderland area, with province location indicated. Time to the most recent common ancestor (TMRCA) of cluster was estimated on 12-Jan-2021.

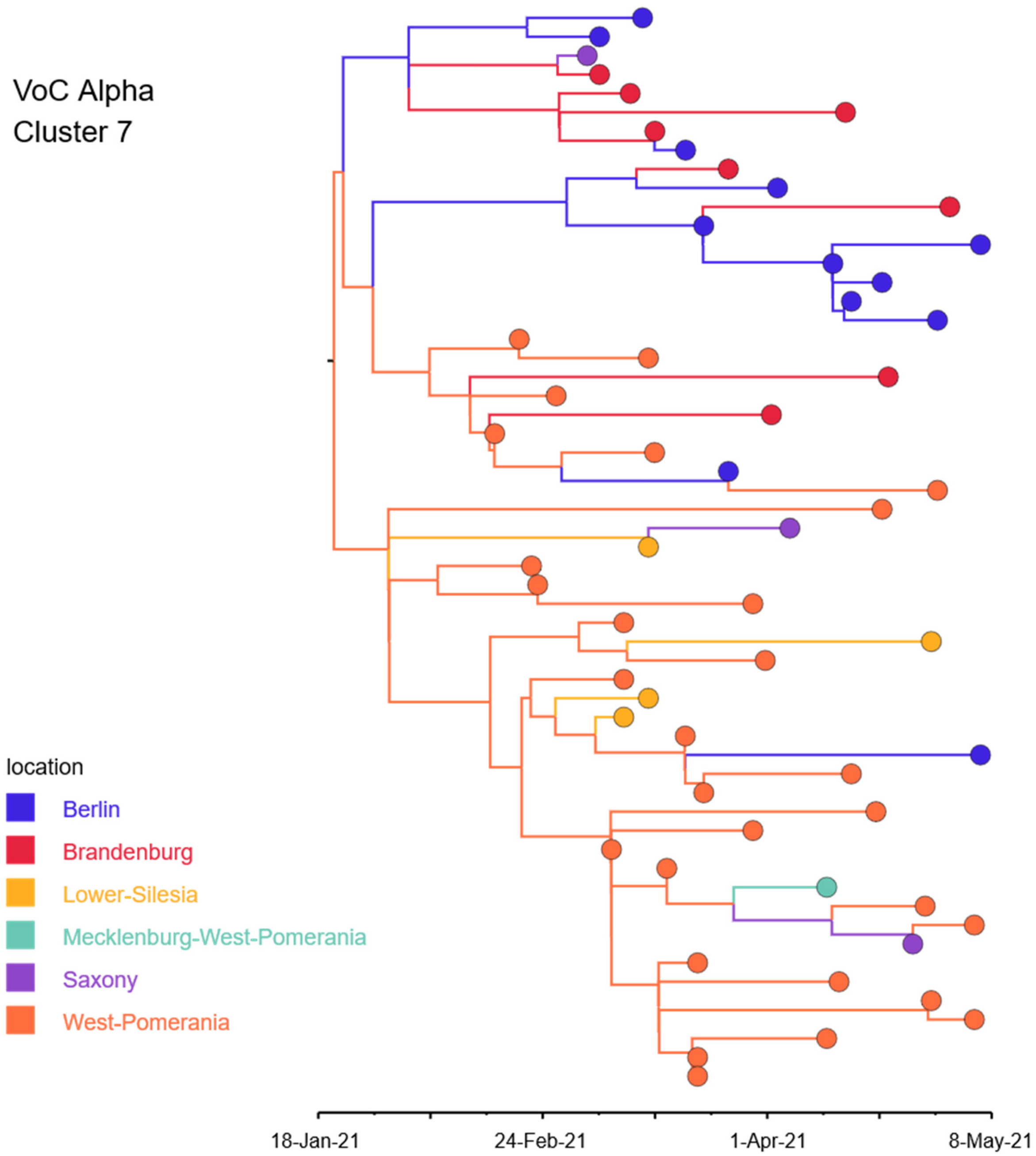

Supplemental Figure S7G. Time-resolved maximum clade credibility phylogeny of the VOC Alpha Cluster 7 among Polish-German borderland area, with province location indicated. Time to the most recent common ancestor (TMRCA) of cluster was estimated on 19-Jan-2021.

Supplemental Table S1. List of non-Alpha Clades identified in the Polish-German frontier area (consisted of three Poland regions and four German states). In **bold**, five Clusters detailed in the present study were marked.

Cluster size	number (percentage) of sequences found in East-Germany	number (percentage) of sequences found in West-Poland	lineage|Clade|
2	1 (50.00%)	1 (50.00%)	B.1.1|20B|
2	1 (50.00%)	1 (50.00%)	B.1.88.1|20A|
2	1 (50.00%)	1 (50.00%)	B.1.351|Beta|
3	1 (33.33%)	2 (66.67%)	B.1.1.374|20B|
3	1 (33.33%)	2 (66.67%)	B.1.177|20E.EU1|
3	1 (33.33%)	2 (66.67%)	B.1.258|20A|
3	2 (66.67%)	1 (33.33%)	B.1.258|20A|
4	3 (75.00%)	1 (25.00%)	B.3|19A|
4	1 (25.00%)	3 (75.00%)	B.1.1.47|20B|
4	2 (50.00%)	2 (50.00%)	B.1.1.277|20B|
5	3 (60.00%)	2 (40.00%)	B.1.1.159|20B|
5	4 (80.00%)	1 (20.00%)	B.1.1.219|20B|
5	4 (80.00%)	1 (20.00%)	A.2|19B|
5	1 (20.00%)	4 (80.00%)	B.1.258.3|20A|
6	5 (83.33%)	1 (16.67%)	B.1.177|20E.EU1|
6	3 (50.00%)	3 (50.00%)	B.1.1.219|20B|
11	8 (72.73%)	3 (27.27%)	B.1.258|20A|
17	3 (17.65%)	14 (82.35%)	B.1.177|20E.EU1|
18	15 (83.33%)	3 (16.67%)	B.1.389|20A|
19	17 (89.47%)	2 (10.53%)	B.1.258|20A|
19	9 (47.37%)	10 (52.63%)	B.1.1.170|20B|
23	14 (60.87%)	9 (39.13%)	B.1|20A|
24	7 (29.17%)	17 (70.83%)	B.1.2|20G|
30	25 (83.33%)	5 (16.67%)	B.1.351|Beta|
38	36 (94.74%)	2 (5.26%)	B.1.1.317|20B|
44	23 (52.27%)	21 (47.73%)	B.1.1.153|20B|
**55**	**31 (56.36%)**	**24 (43.64%)**	**B.1.221|20A|**
56	55 (98.21%)	1 (1.79%)	B.1.177.81|20E.EU1|
63	61 (96.83%)	2 (3.17%)	B.1.351|Beta|
**66**	**23 (34.85%)**	**43 (65.15%)**	**B.1.221|20A|**
**73**	**47 (64.38%)**	**26 (35.62%)**	**B.1.258|20A|**
**119**	**72 (60.50%)**	**47 (39.50%)**	**B.1.258|20A|**
**161**	**93 (57.76%)**	**68 (42.24%)**	**B.1.1|20B|**
581	580 (99.83%)	1 (0.17%)	B.1.177.86|20E.EU1|

Supplemental Table S2. List of VOC Alpha Clades identified in the Polish-German frontier area (consisted of three Poland regions and four German states). In **bold**, seven Clusters detailed in the present study were marked.

Cluster size	number (percentage) of sequences found in East-Germany	number (percentage) of sequences found in West-Poland	Cluster size	number (percentage) of sequences found in East-Germany	number (percentage) of sequences found in West-Poland
2	1 (50.00%)	1 (50.00%)	10	1 (10.00%)	9 (90.00%)
2	1 (50.00%)	1 (50.00%)	10	3 (30.00%)	7 (70.00%)
2	1 (50.00%)	1 (50.00%)	10	7 (70.00%)	3 (30.00%)
2	1 (50.00%)	1 (50.00%)	11	2 (18.18%)	9 (81.82%)
2	1 (50.00%)	1 (50.00%)	11	3 (27.27%)	8 (72.73%)
2	1 (50.00%)	1 (50.00%)	11	7 (63.64%)	4 (36.36%)
3	1 (33.33%)	2 (66.67%)	11	10 (90.91%)	1 (9.09%)
3	1 (33.33%)	2 (66.67%)	13	1 (7.69%)	12 (92.31%)
3	2 (66.67%)	1 (33.33%)	14	1 (7.14%)	13 (92.86%)
3	2 (66.67%)	1 (33.33%)	15	6 (40.00%)	9 (60.00%)
3	2 (66.67%)	1 (33.33%)	16	2 (12.50%)	14 (87.50%)
3	2 (66.67%)	1 (33.33%)	17	2 (11.76%)	15 (88.24%)
3	2 (66.67%)	1 (33.33%)	17	12 (70.59%)	5 (29.41%)
3	2 (66.67%)	1 (33.33%)	17	14 (82.35%)	3 (17.65%)
4	1 (25.00%)	3 (75.00%)	18	3 (16.67%)	15 (83.33%)
4	1 (25.00%)	3 (75.00%)	18	6 (33.33%)	12 (66.67%)
4	1 (25.00%)	3 (75.00%)	18	17 (94.44%)	1 (5.56%)
4	2 (50.00%)	2 (50.00%)	18	17 (94.44%)	1 (5.56%)
4	2 (50.00%)	2 (50.00%)	21	12 (57.14%)	9 (42.86%)
4	3 (75.00%)	1 (25.00%)	21	20 (95.24%)	1 (4.76%)
5	1 (20.00%)	4 (80.00%)	22	1 (4.55%)	21 (95.45%)
5	1 (20.00%)	4 (80.00%)	22	21 (95.45%)	1 (4.55%)
5	3 (60.00%)	2 (40.00%)	23	6 (26.09%)	17 (73.91%)
5	3 (60.00%)	2 (40.00%)	24	1 (4.17%)	23 (95.83%)
5	3 (60.00%)	2 (40.00%)	24	10 (41.67%)	14 (58.33%)
5	3 (60.00%)	2 (40.00%)	27	14 (51.85%)	13 (48.15%)
5	3 (60.00%)	2 (40.00%)	28	24 (85.71%)	4 (14.29%)
6	1 (16.67%)	5 (83.33%)	31	2 (6.45%)	29 (93.55%)
6	3 (50.00%)	3 (50.00%)	35	1 (2.86%)	34 (97.14%)
6	3 (50.00%)	3 (50.00%)	43	4 (9.30%)	39 (90.70%)
6	5 (83.33%)	1 (16.67%)	49	17 (34.69%)	32 (65.31%)
6	5 (83.33%)	1 (16.67%)	**57**	**24 (42.11%)**	**33 (57.89%)**
7	1 (14.29%)	6 (85.71%)	**58**	**14 (24.14%)**	**44 (75.86%)**
7	1 (14.29%)	6 (85.71%)	68	2 (2.94%)	66 (97.06%)
7	3 (42.86%)	4 (57.14%)	74	7 (9.46%)	67 (90.54%)
7	6 (85.71%)	1 (14.29%)	102	100 (98.04%)	2 (1.96%)
8	1 (12.50%)	7 (87.50%)	**118**	**33 (27.97%)**	**85 (72.03%)**
8	1 (12.50%)	7 (87.50%)	**134**	**69 (51.49%)**	**65 (48.51%)**
8	2 (25.00%)	6 (75.00%)	**199**	**51 (25.63%)**	**148 (74.37%)**
8	3 (37.50%)	5 (62.50%)	**412**	**323 (78.40%)**	**89 (21.60%)**
9	8 (88.89%)	1 (11.11%)	**484**	**288 (59.50%)**	**196 (40.50%)**
